# Spatiotemporal regulation of arbuscular mycorrhizal symbiosis at cellular resolution

**DOI:** 10.1093/plcell/koag133

**Published:** 2026-05-11

**Authors:** Tania Chancellor, Gabriel Ferreras-Garrucho, Garo Z Akmakjian, Héctor Montero, Sarah Bowden, Matthew S Hope, Emma Wallington, Samik Bhattacharya, Christian Korfhage, Julia Bailey-Serres, Uta Paszkowski

**Affiliations:** Crop Science Centre, Department of Plant Sciences, University of Cambridge, 93 Lawrence Weaver Road, Cambridge CB3 0LE, United Kingdom; Crop Science Centre, Department of Plant Sciences, University of Cambridge, 93 Lawrence Weaver Road, Cambridge CB3 0LE, United Kingdom; Department of Botany and Plant Sciences and Center for Plant Cell Biology, University of California-Riverside, Riverside, CA 92521-0217, United States; Crop Science Centre, Department of Plant Sciences, University of Cambridge, 93 Lawrence Weaver Road, Cambridge CB3 0LE, United Kingdom; Center for Sustainable Resource Science, RIKEN, Yokohama, Kanagawa 230-0045, Japan; NIAB, 93 Lawrence Weaver Road, Cambridge CB3 0LE, United Kingdom; NIAB, 93 Lawrence Weaver Road, Cambridge CB3 0LE, United Kingdom; NIAB, 93 Lawrence Weaver Road, Cambridge CB3 0LE, United Kingdom; Resolve BioSciences GmbH, Creative Campus Monheim, Creative-Campus-Allee 12, Monheim 40789, Germany; Resolve BioSciences GmbH, Creative Campus Monheim, Creative-Campus-Allee 12, Monheim 40789, Germany; Department of Botany and Plant Sciences and Center for Plant Cell Biology, University of California-Riverside, Riverside, CA 92521-0217, United States; Crop Science Centre, Department of Plant Sciences, University of Cambridge, 93 Lawrence Weaver Road, Cambridge CB3 0LE, United Kingdom; Center for Sustainable Resource Science, RIKEN, Yokohama, Kanagawa 230-0045, Japan

## Abstract

Arbuscular mycorrhizal (AM) symbiosis develops through fungal colonization of root epidermal and cortical cells, culminating in the formation of arbuscules, transient, tree-like intracellular hyphal structures for nutrient exchange. To dissect the complexity of AM establishment in rice (*Oryza sativa*) roots colonized by *Rhizophagus irregularis*, we conducted spatial transcriptomics of plant and fungal genes at single-cell resolution. This revealed differences in transcriptional activity between fungal structures and reprogramming of plant cell-identity markers upon colonization. Furthermore, cells hosting similarly developed arbuscules showed striking transcriptional heterogeneity, suggesting hidden functional diversity at the individual cell level. For stage-resolved profiling of translation, we used AM-stage specific Translating Ribosome Affinity Purification RNA sequencing (TRAP-seq) with promoters active at discrete stages of symbiosis or arbuscule development. This revealed extensive spatiotemporal changes in the ribosome-bound transcript population, including sets of phosphate, nitrogen, and carbon transporters and regulators with specific enrichment and depletion patterns at different stages of arbuscule development. Rice transcripts encoding cell wall biosynthesis genes and defense markers were present in low abundance at early stages but highly abundant at late stages of the arbuscule lifespan, supporting a host-driven shift toward arbuscule termination. Together, these findings highlight the nuanced dynamic regulation of AM symbiosis at the cellular level, refining our understanding of how nutrient exchange and fungal development are coordinated in space and time.

## Teaser

High-resolution tracking of gene activity across cells and over time reveals an exceptionally detailed picture of how plants and arbuscular mycorrhizal fungi coordinate and regulate their interaction.

## Introduction

The symbiotic relationship between plants and arbuscular mycorrhizal (AM) fungi is both ancient and widespread, with over 80% of extant plant species engaging with AM fungi across diverse ecosystems, including domesticated crop species in agricultural settings. The relationship is based on a finely balanced nutritional mutualism. AM fungi greatly increase the uptake of mineral nutrients such as phosphate and nitrogen, whereas the plant provides the fungus with essential carbon, predominantly in the form of fatty acids but also carbohydrates, necessary for the completion of the AM fungal life-cycle (Bravo et al. 2017; Jiang et al. 2017; Keymer et al. 2017; Luginbuehl et al. 2017).

**Figure 1:**
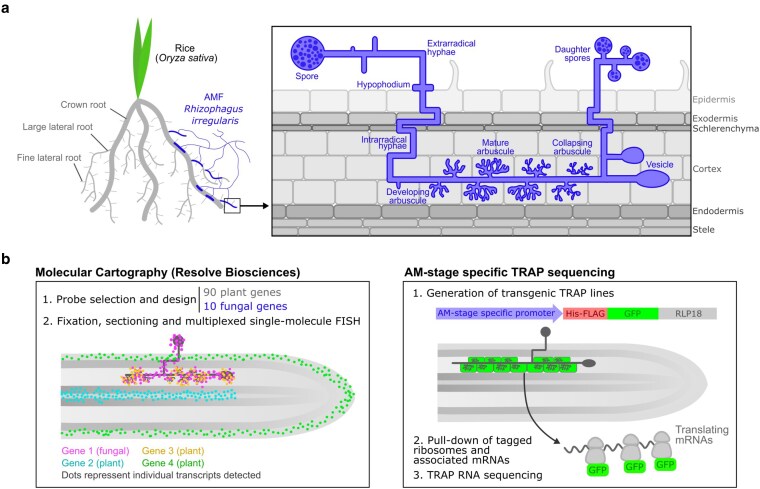
Graphical abstract of the spatio-temporal study of arbuscular mycorrhizal symbiosis between rice and *Rhizophagus irregularis*. a) Simplified representation of the biological system of study, the mutualism between roots of rice (*Oryza sativa*) and the arbuscular mycorrhizal fungus (AMF) *R. irregularis*, with the macroscopic view of the interaction on the left, and the microscopic view of symbiotic fungal structures outside and within the plant root on the right. b) Schematic representation of the high-resolution experimental techniques employed in this study to resolve the spatio-temporal dynamics of AM symbiosis. On the left, Molecular Cartography by Resolve Biosciences, including a simplified mycorrhizal root section with 4 genes detected. FISH, fluorescence in situ hybridization. On the right, Translating-Ribosome Affinity Purification (TRAP) RNA sequencing using AM-stage specific promoters, indicating the structure of the TRAP construct (with a 6x-His-FLAG as well as GFP tags fused to the ribosomal protein RLP18) and hypothetical expression pattern of the construct based on AM-stage specific promoter used.

The establishment of AM symbiosis is tightly coordinated and begins with mutual recognition, followed by the fungus contacting the host root, and initiating a monumental reprogramming of plant cellular architecture that facilitates fungal entry and colonization of the root epidermal and cortical cell layers. Central to the symbiosis is the formation of arbuscules, finely branched fungal structures within root cortical cells. Arbuscules have a short lifespan, forming and collapsing within a few days. They are enveloped by the plant-derived peri-arbuscular membrane (PAM), which is enriched in specialized transporters mediating the bidirectional flow of minerals and fatty acids that underpin the mutualistic interaction. Following arbuscule development, the fungus accumulates host-derived lipids and, in some AM clades, forms storage vesicles inside the root cortex, while reproductive spores develop both inside and outside the root (Fig. 1a) (Smith and Read 2008).

The dynamic and asynchronous nature of fungal root colonization, with co-occurring distinct fungal structures in adjacent plant cells, poses challenges to the study of discrete stages of the association. Similarly, molecular mechanisms that act cell-autonomously to coordinate the rapid formation and turnover of arbuscules remain difficult to identify. As a result, little is known regarding the fine-scale spatiotemporal regulation of AM development. Though vital spatial information has been gained from the use of transcriptional and translational reporters, RNA in situ hybridization, and laser microdissection coupled with RNA-seq, such methods fall short in capturing the heterogeneous complexity of the symbiotic stages across tissues and cell types. Recent advances such as spatial transcriptomics have expanded our ability to interrogate spatial gene expression in AM symbiosis (for recent review see Ferreras-Garrucho et al. 2025). Using single-nucleus sequencing and spatial transcriptomics (Visium, 10X Genomics), Serrano et al. (2024) identified coordinated, stage-specific transcript abundance programs during the symbiosis between *Medicago truncatula* and *Rhizophagus irregularis*. Indeed, the use of Visium spatial transcriptomics enabled plant and fungal transcripts from 1 to 5 plant cells to be captured with each spot, thus a major step toward an increased resolution of transcriptional programs.

In this study, we combined high-resolution spatial transcriptomics with Translating Ribosome Affinity Purification and RNA sequencing (TRAP-seq) to conduct a detailed dissection of the spatial and temporal regulation of AM symbiosis between rice (*Oryza sativa*) and *R. irregularis* (Fig. 1b). Using the Molecular Cartography spatial transcriptomics platform by Resolve Biosciences (a combinatorial smFISH based approach), we targeted a panel of key AM symbiosis genes from plant and fungal targets, providing a spatially defined, dual-species transcript pattern resource at single-cell resolution in plants. We then leveraged TRAP-seq to provide a complementary insight into translational reprogramming across the dynamic AM development (Zanetti et al. 2005; Reynoso et al. 2015, 2022; Zhao et al. 2017). Here, we selected 3 rice promoters known to be activated at different stages of AM colonization. Our AM-stage specific TRAP-seq approach enabled targeted investigations of gene activity at distinct developmental time frames. The high-resolution data presented here provides insights into the spatial expression of plant and fungal AM genes, uncovers significant spatiotemporal repatterning of key nutrient transport and signaling genes, and sheds light on translational reprogramming from arbuscule development through to arbuscule degeneration. Together, our data contribute to an improved understanding of the fine-scale regulation of AM symbiosis in rice.

## Results

### Dual-species spatial transcriptomics at single-cell resolution

To understand how gene regulation supports the dynamic progression of AM symbiosis, we investigated the spatial expression of selected genes across different stages of root colonization using the Molecular Cartography platform from Resolve Biosciences. Here, we curated a panel of 100 targets from rice and the model AM fungus *Rhizophagus irregularis*, comprising 10 fungal genes associated with AM symbiosis and 90 plant genes. The plant set included 22 established cell type-specific markers, 25 genes involved in AM signaling, and genes implicated in the perception, transport, and signaling of nitrogen (11 genes), phosphate (20 genes), and carbon (12 genes) (Table S1). We compared *R. irregularis-*colonized roots (+Ri) with noncolonized control roots (−Ri) harvested at 6 wks post inoculation (wpi). Two independent experiments were performed to strengthen detection of transcript patterns across colonization stages in independent roots of independently grown plants. To capture spatial gene expression across the complex rice root system, our spatial transcriptomics experiments included both crown roots (CRs) and large lateral roots (LLRs).

**Figure 2:**
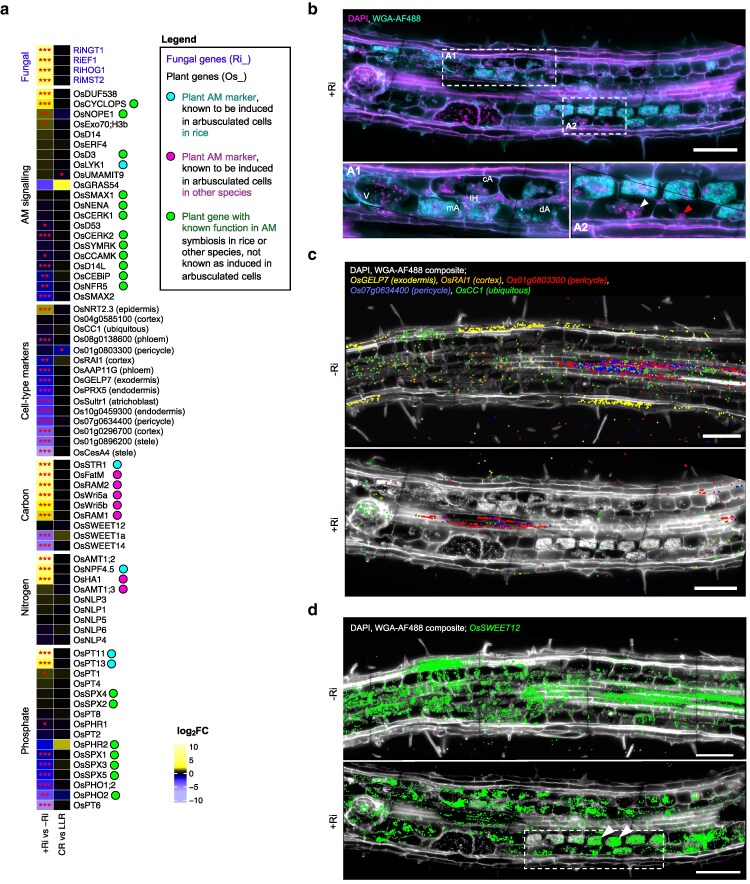
Molecular cartography of *R. irregularis*-colonized rice roots reveals substantial transcriptional repatterning at quantitative and spatial levels. a) Heatmap of log_2_ fold-changes (log_2_FC) for comparisons of transcript spot data between inoculation regimes and root types. Significance levels as determined by DESeq2 shown with asterisks (* for *P*-value <0.05, ** for <0.01, *** for <0.001). Gene names are colored based on species (blue for *R. irregularis*, black for rice). Dots next to rice genes indicate those with previous knowledge regarding their expression pattern (for gene specifically expressed in arbusculated cells, cyan if known for rice, magenta if only known in other species) or involvement in AM symbiosis (green if known function in rice or other species). CR, crown root; LLR, large lateral root; Ri, *Rhizophagus irregularis* inoculation. b) Composite image of DAPI (magenta) and WGA-AF488 (cyan) of an *R. irregularis*-inoculated (+Ri) large lateral root (LLR) section for visualization of nuclei, cell boundaries and fungal structures. The composite is placed on a black background, and image is reused in Figs. 2c, 2d, 3a, 3d, 4b, 4e, Figure S6, S7. Close-up A1 showcases all fungal structures visualized: intraradical hyphae (IH), arbuscules at distinct development stages (developing, dA; mature, mA; and collapsing, cA), and vesicles (V). Close-up A2 showcases the difference between plant (red), and fungal (white arrow) nuclei. c) Image-transcript overlays showing spatial expression of cell-type specific markers in a −Ri and a +Ri section, both of large lateral roots. −Ri image is reused in Figures S2a, S4, S7. d) Image-transcript overlays highlighting spatial patterning of *OsSWEET12* in a −Ri and a +Ri section, both of large lateral roots. DAPI/WGA-AF488 composite in white, placed on a black background, different colors correspond to independent transcripts, each spot corresponds to 1 detected transcript. Dotted square and arrows highlight transcript abundance heterogeneity between arbuscules at the same developmental stage. Scale bar, 100 μm.

Following 100-plex single molecule fluorescent in situ hybridization (smFISH, Molecular Cartography, Resolve Biosciences), probe performance and section quality were assessed, and samples/probes were filtered before proceeding with data analyses. Probes with less than an average of 20 transcripts per section in both noncolonized and mycorrhizal conditions were removed from further analysis, leaving a total of 74 probes (70 plant, 4 fungal) (Fig. 2a, Table S1). Of 30 sections, 20 were of sufficiently high-quality, leaving a total of 6 sections from experiment 1 (2 noncolonized, 4 mycorrhizal), and 14 sections from experiment 2 (6 noncolonized, 8 mycorrhizal) (Table S2). To scrutinize transcript abundances across all samples, we generated a gene count matrix from the transcript spot data, by quantifying the total number of transcripts of each gene detected for each section (Table S3). Principal Component Analysis (PCA) revealed a distinct separation of samples based on mycorrhizal status along PC1, which accounted for 68% of the variance. Notably, PC2 captured a modest variation (9%) within mycorrhizal samples across the 2 experiments (Figure S1a). This separation is most likely driven by a subset of 24 transcripts, predominantly low-abundance genes, that exhibit high variability in their expression levels, even among biological replicates within the same experiment (Figure S1b, c, Table S3). Transcript abundance did not differ significantly between CR and LLR root types except for 2 genes (the vascular markers *Os01g0803300* and *OsUMAMIT9*), whereas more than half of the set, 48 out of 74 genes, were differentially abundant between *R. irregularis*-colonized and mock samples across root types (Fig. 2a, Figure S1c). To focus on +Ri vs −Ri differences and to maximize the range of cell types captured, CR and LLR samples have been considered together for the remainder of the study, unless otherwise mentioned.

Composite imaging with DAPI and WGA-AF488 revealed a clear blueprint of plant and fungal organization, highlighting nuclei and AM fungal structures, respectively (Fig. 2b). DAPI staining confirmed earlier observations that fungal nuclei are absent from fine arbuscule branches, but present in the arbuscule trunk (Bianciotto et al. 1995), where we also found them during arbuscule senescence, and additionally throughout intraradical hyphae, and at high abundance in vesicles (Fig. 2b). To explore the dual-species Molecular Cartography approach, we assessed the spatial expression of cell-type marker genes across all sections. A total of 22 cell-type marker probes were selected from a list validated by Zhu et al. (2025) based on published single-cell datasets in rice (Liu et al. 2021; Wang et al. 2021; Zhang et al. 2021). Of these, 15 markers yielded sufficient transcript counts (>20 transcripts per section) and were carried forward for detailed analysis. In noncolonized samples, 10 of the 15 markers exhibited spatial expression patterns consistent with their expected tissue localization (Table S1). For example, the exodermal marker *GDSL ESTERASE/LIPASE PROTEIN 7* (*OsGELP7*), the cortical marker *REDOX ASSOCIATED INTERMEDIATE 1* (*OsRAI1*), and 2 uncharacterized phloem-enriched translated mRNAs (*Os07g0634400* and *Os01g0803300*) (Reynoso et al. 2022) aligned closely with their predicted distributions (Fig. 2c). In contrast, the phloem-expressed *AMINO ACID PERMEASE 11G* (*OsAAP11G*), the atrichoblast-expressed nitrate transporter *OsNRT2.3* and the endodermal marker *Os10g0459300* displayed nonspecific spatial expression patterns (Figure S2a). Their diverging expression patterns from previous spatial and single-cell transcriptomics datasets (Liu et al. 2021; Wang et al. 2021; Zhang et al. 2021; Zhu et al. 2025) might be due to the distinct sample type. Whereas prior studies analyzed embryonic root tips grown in hydroponic or plate conditions, our analysis focused on the mature zone of non-embryonic roots cultivated in sand. This highlights the importance of identifying robust cell-type markers across developmental ages, root zones, root types and substrates.

Interestingly, the transcript abundance of most cell-type markers (11/15) was reduced in mycorrhizal samples (Fig. 2a, c), suggesting a profound reprogramming of the root tissue in response to AM fungal colonization. As the highly expressed *Os07g0634400* and *Os01g0803300* retained their expected spatial patterning in colonized roots (Fig. 2c), and the ubiquitously expressed *CYTOCHROME C1* (*OsCc1*) was not downregulated (Fig. 2a), the reduction of cell-type marker transcripts is unlikely to be a technical artifact and instead may indeed reflect a biological consequence of AM symbiosis. It seems therefore plausible that intraradical fungal colonization markedly affects cell-type identity, or at least expression of cell-identity markers. Also notably, although overall transcript levels for the sugar transporter *OsSWEET12* did not differ significantly between conditions (Fig. 2a), spatial mapping showed redistribution in colonized roots, where expression shifted to arbuscule-containing cortical cells (Fig. 2d, Figure S2b). Therefore, AM colonization can reprogram the root transcriptome either to alter transcript abundance while retaining spatial patterning, as with cell-identity markers, or altering spatial distribution without affecting overall expression levels, as in the case of *OsSWEET12*.

**Figure 3:**
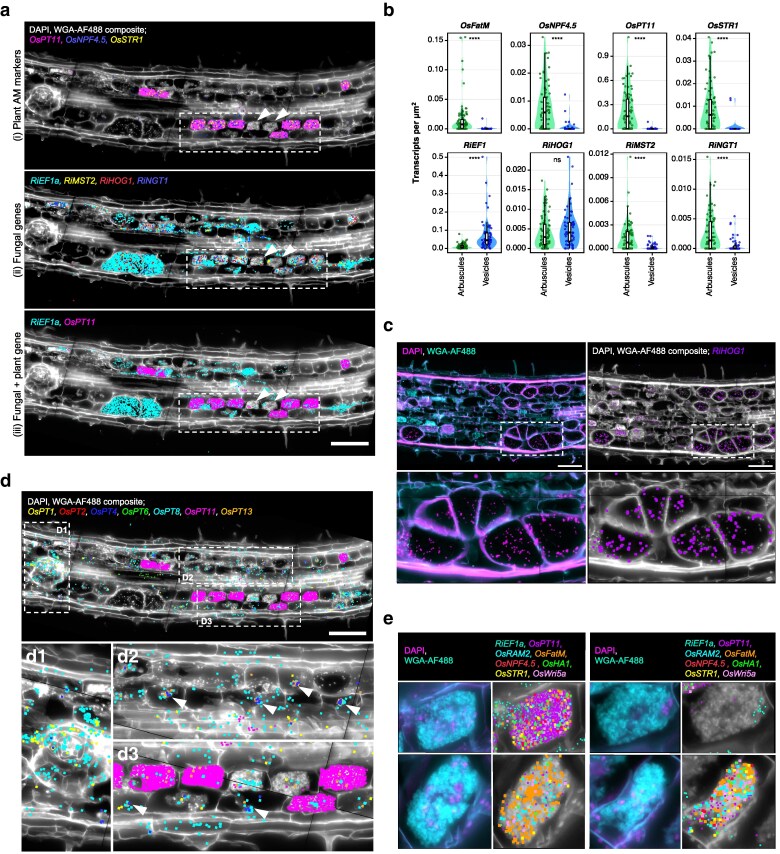
Molecular cartography highlights heterogeneity in fungal and plant transcript abundances across mycorrhizal structures at the same or distinct developmental stages. a) Image-transcript overlays comparing spatial expression of plant and fungal transcripts in an inoculated (+Ri) large lateral root (LLR) section: (i) plant AM marker genes, (ii) fungal genes, (iii) 1 plant AM marker and 1 fungal gene. DAPI/WGA-AF488 composite in white, different colors correspond to independent transcripts, each spot corresponds to 1 detected transcript. Dotted square and arrows highlight transcript abundance heterogeneity between arbuscules at the same developmental stage. b) Quantification of transcript abundance in distinct *R. irregularis* colonization structures in Molecular Cartography +Ri sections. Vesicles and arbusculated-cells from several sections were manually segmented in ImageJ and transcript spot counts for all transcripts in each individual structure quantified. Each graph corresponds to 1 transcript, name above. Each spot corresponds to the transcript per μm^2^ value for an individual structure (arbuscule or vesicle), *n* = 96 for arbuscules, *n* = 67 for vesicles. *P*-value of *T*-student test between structures indicated in each graph. c) Association between fungal nuclei and transcripts in vesicles of in an inoculated (+Ri) crown root (CR) section. Left panels show composite image of DAPI (magenta) and WGA-AF488 (cyan) for visualization of nuclei, cell boundaries and fungal structures. Right panel shows image-transcript overlays for fungal genes; DAPI/WGA-AF488 composite in white, different colors correspond to independent transcripts, each spot corresponds to 1 detected transcript. Image is reused in Figures S2b, S3, S6, S7 and S8. First row for full section, second row for magnified vesicles, location indicated on first row. d) Image-transcript overlays showing spatial expression of the 7 most abundant plant phosphate transporters in an inoculated (+Ri) large lateral root (LLR) section. Close-ups D1 to D3 highlight differing spatial detection patterns in diverse cell types: D1 in emerging lateral root, D2 and D3 for arbuscules at distinct developmental stages and non-colonized cortical cells. Arrows highlight clusters of *OsPT4*, *OsPT8* and *OsPT13* transcripts around plant nuclei of non-colonized and collapsing arbuscule-containing cells. DAPI/WGA-AF488 composite in white, different colors correspond to independent transcripts, each spot corresponds to 1 detected transcript. Scale bar, 100 μm. e) Image-transcript overlays demonstrating transcript abundance heterogeneity of mature arbuscules. Arbuscules extracted from various inoculated (+Ri) crown and large lateral root sections. Left panels show composite image of DAPI (magenta) and WGA-AF488 (cyan) for visualization of nuclei, cell boundaries and fungal structures. Right panel shows image-transcript overlays for fungal genes; DAPI/WGA-AF488 composite in white, different colors correspond to independent transcripts, each spot corresponds to 1 detected transcript. All composite images are placed on a black background.

Next, we examined detection of fungal transcripts and plant AM markers, to validate the capacity of this technology for dual-species spatial transcriptomics. These included the nutrient transporters *PHOSPHATE TRANSPORTER 11* (*OsPT11*), *NITRATE/PEPTIDE FAMILY TRANSPORTER 4.5* (*OsNPF4.5*), and *STUNTED ARBUSCULE 1* (*OsSTR1*), which were specifically localized to arbuscule-containing cells in mycorrhizal roots, as previously shown in rice (Gutjahr et al. 2008, 2012; Wang et al. 2020) (Fig. 3ai, b). The fungal transcripts elongation factor *RiEF1a*, monosaccharide transporter *RiMST2*, *N*-acetylglucosamine transporter *RiNGT1* and mitogen-activated protein kinase *RiHOG1*, closely tracked the distribution of fungal structures (vesicles, hyphae, arbuscules) in colonized roots (Helber et al. 2011; Nadal et al. 2017; Wang et al. 2023) (Fig. 3aii). The fungal marker *RiEF1a* had striking transcript abundance within vesicles. Manual segmentation of arbuscules and vesicles from diverse sections followed by transcript spot quantification showed *RiEF1a* transcripts were significantly more abundant per unit area in vesicles than in arbuscules (Fig. 3b). Conversely, *RiMST2* and *RiNGT1* were more abundant in arbuscules, while *RiHOG1* was similarly abundant in both (Fig. 3b). This highlights that the *R. irregularis* mycelium has distinct domains of expression or transcript accumulation inside plant roots. Notably, within vesicles, in particular *RiHOG1* transcripts co-localized with fungal nuclei (Fig. 3c). This nuclear association suggests the existence of different subcellular domains where transcripts accumulate, and that vesicles are not simply passive storage bodies but transcriptionally and translationally—as suggested by the high abundance of *RiEF1a* transcripts—dynamic compartments, with active expression of certain genes concentrated around nuclear domains. Importantly, we were able to successfully capture transcripts of the 2 species within single plant cell spaces, such as for instance *OsPT11* and *RiEF1a* within arbusculated cells at the fine-branching stage (Fig. 3aiii). These patterns were observed in several independent mycorrhizal roots (Figure S3), and were completely absent from non-mycorrhizal sections (Figure S4).

As our panel included most high-affinity phosphate transporters in rice, involved in both direct (*OsPT1*, *PT2*, *PT4*, *PT6*, *PT8*) and AM-mediated (*OsPT11*, *PT13*) Pi uptake (Paszkowski et al. 2002; Ai et al. 2009; Jia et al. 2011; Sun et al. 2012; Yang et al. 2012), a targeted investigation of their expression patterns was conducted. The symbiotic phosphate transporters *OsPT11* and *OsPT13* were significantly induced in mycorrhizal roots (Fig. 2a). *OsPT11* expression was restricted to arbuscule-containing cells at the mature or fine-branching stage, whereas *OsPT13* was also detected in cells containing collapsing arbuscules (Fig. 3d, Figure S3). Interestingly, non-symbiotic PTs had distinct expression patterns between +Ri and −Ri roots. *OsPT6* was largely confined to the stele regardless of colonization (Fig. 3d, Figures S3, S4), but was significantly downregulated in mycorrhizal roots (Fig. 2a). Conversely, *OsPT1* was generally significantly induced in +Ri roots (Fig. 2a), highly abundant at the base of emerging laterals and stele (Fig. 3d, Figures S3, S4). *OsPT8* was the most abundant and expressed widely across most cell types in both −Ri and −Ri roots, including in arbuscule-containing cells (Fig. 3d, Figure S4). Furthermore, in mycorrhizal roots, *OsPT8*, as well as the less abundant *OsPT4* and *OsPT13*, showed a striking sub-cellular spatial association with plant nuclei in cells containing collapsing arbuscules and in non-colonized cortical cells (Fig. 3d, arrows). Based on *OsPT13* expression, alongside being located next to arbusculated cells, these uncolonized cells were considered to be the final stage of arbuscule collapse. Together, these patterns highlight diverse spatial domains and colonization-dependent regulation of phosphate transporters, extending beyond a simple symbiotic versus non-symbiotic division.

Strikingly, some arbuscules that were at the fully developed fine-branching stage did not express classical markers of the nutrient exchange, such as *OsPT11*, *OsSTR1* and *OsNPF4.5*, and had reduced abundance of fungal transcripts (Fig. 3a, arrows). However, other transcripts such as *OsSWEET12* were detected in these same cells (Fig. 2d), therefore this is unlikely to be a technical artifact. This phenomenon of morphologically indistinguishable arbuscules displaying highly divergent expression signatures was observed repeatedly in independent sections (Figure S3), even within the same file of cortical cells (Fig. 3a), and extended to most transcripts found to be arbuscule-specific or induced (Fig. 3e). This heterogeneity could be partially explained by the inherent transcriptional dynamics during arbuscule development. However, the substantial variation in expression profiles among morphologically similar arbuscules, which do not clearly align with transitional (“developing-like” or “collapsing-like”) states, suggests additional layers of regulation beyond simple developmental progression. This could reflect highly dynamic transcriptional changes within narrow developmental windows, cell-autonomous regulatory processes responsive to local cellular context (such as nutrient status, signaling flux, and host regulatory inputs), or a combination of both temporal and spatial factors. Together, these observations suggest that arbuscules are not uniform structures but display a degree of “individuality’ in their molecular profiles.

### Single-cell clustering and colocalization analyses

**Figure 4:**
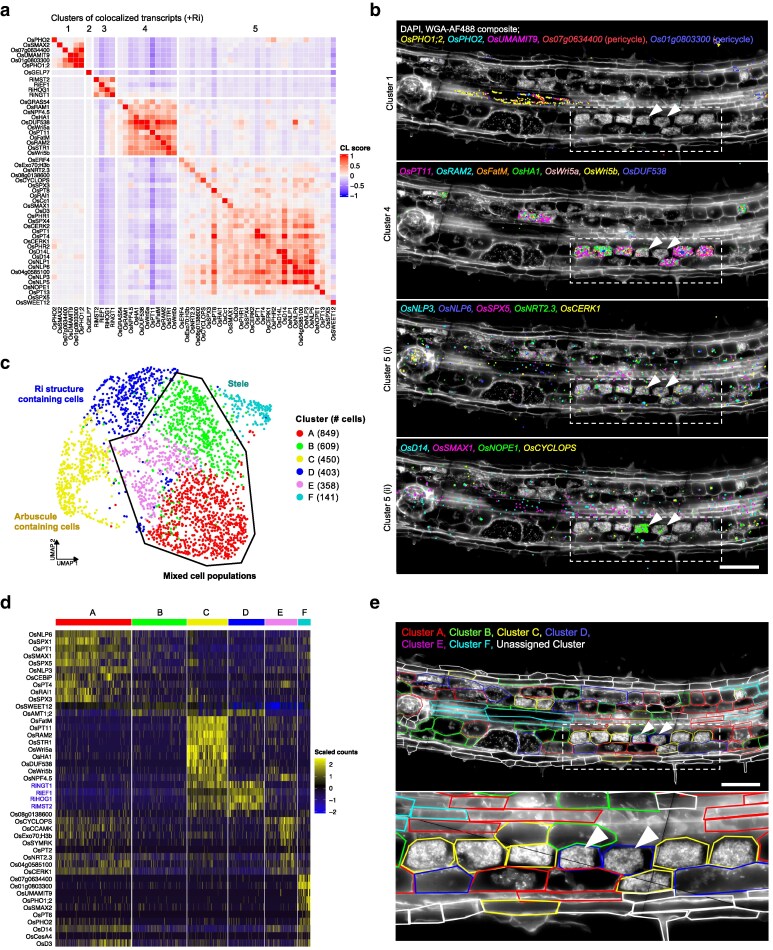
Co-localization and cell segmentation analyses for molecular cartography mycorrhizal samples reveal distinct clusters of co-localized transcripts and cell populations. a) Colocalization heatmap of transcript colocalization scores for +Ri sections. Colocalization scores between each gene pair, quantifying the frequency of finding transcripts in spatial proximity at pixel resolution, were determined using the Polylux plugin (Resolve Biosciences) in ImageJ. Note that the colocalization matrix is asymmetric (*x*-axis represents the starting transcript searching its neighborhood; *y*-axis represents transcripts found in that search). Genes with >0.2 self-co-localisation scores were used for analysis and subjected to hierarchical clustering. Distinct clusters are indicated by thicker boundaries between genes. Color scale represents the transcript colocalization (CL) score. b) Image-transcript overlays highlighting the spatial expression of key marker genes in selected colocalization clusters in an inoculated (+Ri) large lateral root (LLR) section. DAPI/WGA-AF488 composite in white, placed on a black background, different colors correspond to independent transcripts, each spot corresponds to 1 detected transcript. Dotted square and arrows highlight transcript abundance heterogeneity between arbuscules at the same developmental stage. Scale bar, 100 μm. c) UMAP projection of segmented cells, color indicates the cluster (A to F) assigned to each cell by *Seurat* single-cell analysis, number of cells in each cluster indicated in the legend. d) Heatmap of normalized scaled transcript counts for each cell (columns) for the top 10 marker genes (rows) in each cell cluster (column sections), and their relative expression level compared with other clusters. e) Assignment of clusters to segmented cells based on Seurat single-cell analyses in an inoculated (+Ri) large lateral root (LLR) section. DAPI/WGA-AF488 composite in white, placed on a black background, segmented cells in colored lines, distinct colors for separate clusters (“Unassigned cluster” used for cells filtered out during quality control in single-cell analysis). Dotted square (magnified below) and arrows highlight transcript abundance heterogeneity between arbuscules at the same developmental stage. Scale bar, 100 μm.

To investigate the spatial integration of AM symbiosis genes, we calculated transcript colocalization (CL) scores for all 74 targets, which quantify the degree to which different transcripts occupy nearby spatial locations. Low-abundance genes that showed poor self-CL (score < 0.2) were excluded, leaving 51 high CL quality genes for +Ri, and 49 for −Ri samples. These were subjected to hierarchical clustering using their CL scores to determine clusters of highly co-localized transcripts. Despite transcript counts being generally low in noncolonized samples, 4 distinct clusters emerged, largely reflecting rice cell types (cortex, stele or vascular tissue, and exodermis; Figure S5). By contrast, colonized samples displayed a markedly altered transcriptional landscape, with 5 distinct clusters that reflected a profound reorganization of spatial expression patterns across root tissues (Fig. 4a, b).

Cluster 1 comprised vascular-associated targets, including *Os01g0803300*, *Os07g0634400*, xylem-loading phosphate transporter *OsPHO1;2*, phosphate-starvation regulator *OsPHO2*, and amino-acid transporter *OsUMAMIT9* (Fig. 4a, b). While exodermal markers clustered together in the mock condition, their reduced abundance in colonized roots left *OsGELP7* as the sole representative in cluster 2 (Figs. 2c, 4a), possibly reflecting symbiosis-induced reprogramming of outer root layers. Cluster 3 contained fungal genes which formed a distinct group with no strong associations to rice targets, conceivably because of transcript accumulation in all of the fungal structures, not just arbuscules (Figs. 3Aii, 4a). Cluster 4 included genes that were specifically expressed in mature arbusculated cells, mostly involved in nutrient transfer, including the previously mentioned known rice AM markers *OsPT11*, *OsSTR1* and *OsNPF4.5*, as well as genes known to be arbuscule-specific in other species, such as the symbiotic H^+^-ATPase *OsHA1*, the lipid biosynthetic enzyme *OsFatM* and the transcriptional regulators *OsRAM2* and *OsWRI5a/b* (Krajinski et al. 2002; Gobbato et al. 2013; Bravo et al. 2017; Luginbuehl et al. 2017) (Fig. 4a, b). This group also included *OsDUF538*, a gene known to be induced by AM colonization and conserved in AM-host species (Bravo et al. 2017; Sgroi et al. 2024), but with unknown spatial distribution of its transcripts. The arbuscule-specific expression pattern of *OsDUF538* and high co-localization with nutrient-related transcripts suggests a role during this intimate stage of the interaction (Fig. 4a, b). Cluster 5 is the largest and most varied, containing AM and phosphate signaling genes (*OsSPXs*, *OsCERK1*, *OsD14*, *OsSMAX1*), all phosphate transporters except *OsPT11*, and sugar transporter *OsSWEET12*, harboring more varied spatial patterns, including genes whose spatial distribution shifts during colonization such as *OsSWEET12* and *OsPT8* (Figs. 2d, 3d, 4a, b).

While transcript CL identified groups of genes with shared spatial patterns across tissues, this approach does not directly link expression profiles to individual cells. To resolve how these patterns map onto discrete cellular states and AM developmental stages, we next performed single-cell segmentation of colonized roots (Figure S6), generating an expression matrix of 3,464 cells (Table S4). Vesicles were treated as single “cells’. Six cell clusters were identified, which could be mapped back to their spatial location to identify the cell-type and AM developmental stage, facilitating annotation (Fig. 4c to e). Cluster C cells were the most consistent in both transcript abundance and AM developmental stage, with clear expression of arbuscule-localized plant genes as well as fungal transcripts, and with the majority of cells containing arbuscules, reflecting an intimate symbiotic state. Cluster D encompassed cells containing fungal structures (intraradical hyphae, vesicles) and showed high expression of fungal genes (Fig. 4c to e). Cluster D also showed surprising abundance of ammonium-transporter *OsAMT1;2*, which upon closer observation was present within fungal structures in similar pattern to other fungal transcripts (Figure S7). This perplexing observation cannot easily be explained but could be due to a technical artifact, such as plant probes recognizing a fungal transcript for which the encoding gene is currently missing from the genome sequence annotation. Cluster F grouped mostly stele-associated cells, aligning with transcript CL cluster 1 (Fig. 4c to e). However, clusters A, B and E cells were highly variable in terms of transcript abundance and AM development. Cluster A was enriched for symbiosis-related signaling genes (*OsSMAX1, OsNLP6, OsSPXs*), cluster B cells did not express any distinct marker gene, and cluster E cells displayed reduced *OsSWEET12* but elevated *OsCYCLOPS* and *OsNPF4.5*. More surprisingly, these clusters contained cells at different stages of colonization, ranging from uncolonized cortical cells, cells with developing/collapsing arbuscules, as well as uncolonized cells in the stele (Fig. 4c to e).

Therefore, while single-cell analysis of spatial transcriptomics data enabled the classification of distinct cell populations, it was not sufficient to accurately predict the AM developmental stage of individual cells. This can be directly linked to the previous observation that arbuscules at an equivalent developmental stage displayed highly divergent expression signatures (Fig. 3e), which results in arbusculated cells at the same stage of maturity being classified in distinct cell clusters (Fig. 4b, e, Figure S8). This phenomenon of “arbuscule individuality” appears more prominent than, and thus masks, the dynamic transcriptional regulation due to arbuscule development, at least within our limited probe-set. To further explore this heterogeneity at the single-cell level, we performed targeted single-cell analysis on 96 manually segmented mature arbusculated cells (from Fig. 3b). Three distinct clusters emerged (Figure S9): cluster A1 (termed as “canonical” arbuscule) expresses established arbuscule-specific genes including nutrient transporters (*OsPT11*, *OsNPF4.5, OsSTR1*) and AM regulators (*OsWri5a*, *OsWri5b*, *OsFatM*); cluster A3 (“non-canonical”) lacks these canonical markers but instead expresses genes not previously characterized as arbuscule-specific (*OsSWEET12*, *OsAMT1;2*, *OsPT8*); and cluster A2 (“mixed”) shows expression from both gene sets. All clusters show similar expression of fungal genes. Notably, substantial cell-to-cell variability persists within clusters, particularly in the non-canonical A3 cluster, with some arbusculated cells displaying highly unique expression profiles that reinforce the concept of arbuscule individuality.

### AM-stage specific TRAP-seq for targeted investigation of translational responses across developmental stages of AM symbiosis

**Figure 5:**
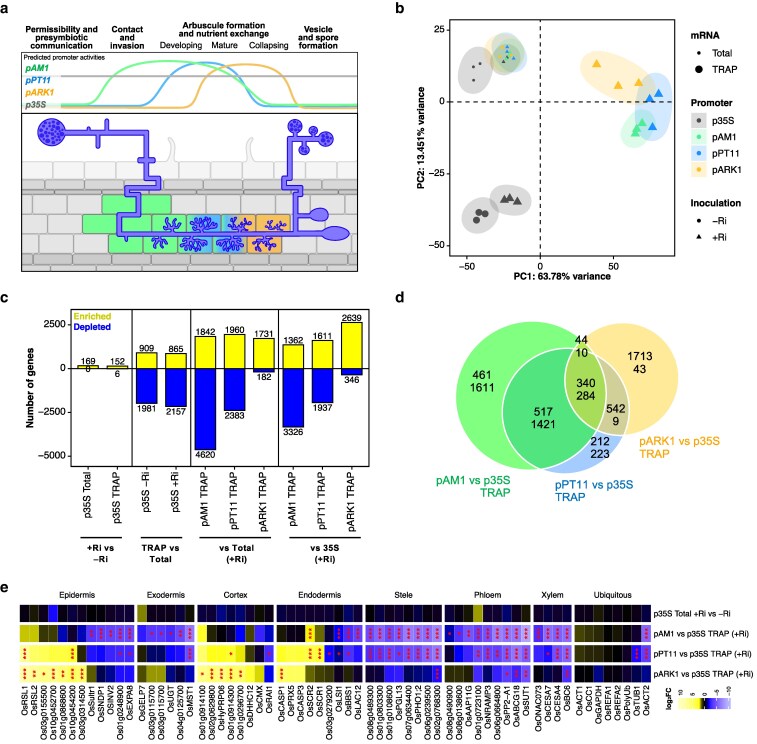
AM-stage specific TRAP-seq shows dynamic translational reprogramming across AM development. a) Schematic representation of predicted activities through root cell layers and AM development of the promoters used for AM-stage specific TRAP. b) Principal component analysis (PCA) visualization for all samples in the dataset, using the top 1,000 variable features from normalized variance-stabilized-transformed (VST) gene counts. PC1 and PC2 displayed as explaining a combined 77.2% of the variance. Color based on genotype, that is, promoter driving TRAP construct. Size based on total vs TRAP mRNA. Shape based on inoculation. Best fit ellipses for each treatment group were created using the Khachiyan algorithm from the *ggforce* package in R (Pedersen 2025). c) Number of differentially captured genes in all relevant comparisons between conditions. Positive yellow bars for upregulated genes, negative blue bars for downregulated genes. d) Venn diagram showing overlaps between DEGs for each AM inducible promoter vs *p35S* TRAP samples, first number corresponds to upregulated genes, second number to downregulated genes. e) Heatmap of log_2_ fold-changes (log_2_FC) for a selection of comparisons of interest, for a selection of cell-type specific marker genes (full comparison list and complete set of marker genes on Figure S13). Significance levels as determined by DESeq2 shown with asterisks (* for *P*-value <0.05, ** for <0.01, *** for <0.001). Genes were subjected to hierarchical clustering within each group.

To address the challenge of capturing dynamic gene regulation across continuously shifting AM or also arbuscular developmental stages, we applied AM-stage specific TRAP-seq. TRAP-seq captures transcripts that are associated with ribosomes containing an epitope-tagged Ribosomal Protein L18/u18 produced from a chimeric gene driven by a cell-type or conditional promoter (Mustroph et al. 2009b). By isolating ribosome-bound transcripts, TRAP-seq provides a direct proxy of active translation (Lee and Bailey-Serres 2019). Three rice promoters known to be activated at different stages of AM colonization were used to construct TRAP lines (Fig. 5a). The *ARBUSCULAR MYCORRHIZAL SPECIFIC MARKER 1* (*OsAM1*) encodes a putative type III peroxidase PRX53, and is highly induced during mycorrhizal colonization (Güimil et al. 2005). *OsAM1* transcripts accumulate in cells containing small arbuscules and in cells flanking intercellularly growing hyphae (Gutjahr et al. 2008), and thus the *OsAM1* promoter is already active during early colonization, and remains active as new infection events arise during the asynchronous progression of symbiosis. The well-characterized *OsPT11* encodes an AM-specific phosphate uptake transporter which is expressed in the PAM surrounding fine-branched arbuscules, hence representing a “mid” colonization reporter marking the early-mature arbuscule development stages (Kobae and Hata 2010). Finally, the *ARBUSCULAR RECEPTOR-LIKE KINASE 1* (*OsARK1*) encodes a receptor-like kinase (RLK) which regulates AM fungal fitness and plays a crucial role in symbiotic maintenance during post-arbuscule development. Translational reporter lines demonstrate that OsARK1 is localized to the PAM surrounding fine branches of mature and collapsing arbuscules, therefore representing a mid-late colonization reporter (Roth et al. 2018).

The 3 AM-stage specific TRAP lines (*pAM1:TRAP, pPT11:TRAP, pARK1:TRAP*) were investigated alongside a constitutive TRAP line generated using the *CaMV35S* promoter (*p35S:TRAP*) (Reynoso et al. 2022). To ensure uniform and extensive colonization at an early time point of 3 wks-post-inoculation, a nurse inoculation system was employed. For each line, both the TRAP fraction (ribosome-bound mRNAs) and the total mRNA pool were sequenced. Comparison of total and TRAP profiles from the constitutive *p35S:TRAP* line, grown under mycorrhizal conditions, established a baseline for differences between total and ribosome-associated mRNA populations in colonized roots. Applying the same analysis to the AM-stage specific TRAP lines revealed additional changes reflecting both translational regulation and the enrichment of specific cell types targeted by each promoter. Finally, by comparing the TRAP profiles of each AM-stage specific line with that of the constitutive *p35S:TRAP* line, we wished to identify translational signatures specifically associated with distinct stages of AM development.

PCA revealed clear separation of samples according to mycorrhizal status (+/−Ri), mRNA source (total or TRAP), and promoter used for TRAP lines (*pAM1, pPT11, pARK1, p35S*) (Fig. 5b). Along PC2 (13.5% variance), total versus TRAP samples of the constitutive *p35S:TRAP* line were separated, reflecting differences between the transcriptome and translatome in whole tissue. PC1 (63.8% variance) primarily distinguished inoculated from non-inoculated samples, across both total and TRAP mRNA in the *p35S:TRAP* line, as well as between promoters driving TRAP expression. This axis therefore captured transcriptional and translational alterations associated with AM symbiosis and its cell populations. These patterns demonstrate that TRAP was effective in both constitutive and AM-stage contexts, resolving promoter-specific translatomes distinct from the bulk transcriptome. Consistent with this, normalized gene counts showed strong enrichment of *OsAM1*, *OsPT11* and *OsARK1* in the TRAP fractions of their respective lines, an order of magnitude greater than in the *p35S:TRAP* line, confirming targeted enrichment (Figure S10a, Table S5).

Differential abundance analysis conducted on the total mRNA profiles of *p35S:TRAP* identified 169 upregulated genes in response to AM colonization (+Ri vs −Ri) (Fig. 5c) consistent with the general predominance of upregulated over downregulated genes in mycorrhizal systems (Fiorilli et al. 2013; Rich et al. 2017; Vangelisti et al. 2018; Sgroi et al. 2024). These almost completely overlapped with published bulk RNA-seq datasets (Figure S10b). The relatively modest number of DEGs suggests that sampling captured an overall early stage of colonization, as expected at 3 wpi. The same comparison performed on TRAP mRNA revealed 152 upregulated and 6 downregulated genes, with substantial overlap with the total mRNA set (109 shared) but also fraction-specific responses; for example, *OsNOPE1* was induced only in total mRNA, whereas *OsPT13* was specific to the TRAP fraction (Figure S10b). Finally, direct comparison of TRAP and total mRNA pools within each colonization state revealed extensive differences (2,890 and 3,022 DEGs in −Ri and +Ri roots, respectively), of which one-third was dependent of colonization and included translational shifts in strigolactone/karrikin signaling genes, such as *OsD53* and *OsSMAX1* (altered only +Ri), or *OsD53L* and *OsSMXL2* (altered only −Ri) (Figure S10c). Collectively, these results show that AM symbiosis affects both transcriptional and translational regulation, largely through parallel expression changes but with distinct cases of translational control, consistent with observations in other biotic interactions (Traubenik et al. 2020, 2021).

We next compared the TRAP mRNA profiles of each AM-stage-inducible line with (i) its corresponding total mRNA profile and (ii) the TRAP profile of the constitutive *p35S:TRAP* line. Both comparisons revealed significant enrichment of suites of transcripts, with comparable overall magnitudes of change, though enrichment tended to be slightly higher relative to the total mRNA profiles, except in the *pARK1* line (Fig. 5c). This is consistent with both comparisons capturing transcripts enriched or depleted in the specific cell populations targeted, while the contrast with total mRNA additionally reflects translatome versus transcriptome differences. Importantly, the number of genes differentially captured in AM-stage specific TRAP samples relative to *p35S* was an order of magnitude greater than in the +Ri vs −Ri comparison, indicating a strong enrichment of colonized cell populations. This is further supported by bulk mycorrhizal transcriptional response being largely recapitulated in the *pAM1* versus *p35S* TRAP comparison, with 147 out of 169 genes recovered (Figure S10d). Moreover, when comparing with bulk RNA-seq data at a later co-cultivation time-point of 6 wks, with higher colonization levels (Das et al. 2022), a further 529 genes transcriptionally activated by AM colonization, not captured in our early bulk dataset, were significantly enriched in the *pAM1:TRAP* samples (Figure S10d). This further supports the specific capture of colonized cell populations, as the TRAP profile recapitulates the bulk transcriptomic response characteristic of roots with higher colonization levels. Notably, beyond this established AM transcriptional response, more than half of the genes enriched for *pAM1:TRAP* samples, as well as a substantial down-regulation of genes absent from the total mRNA comparison, were uniquely captured by AM-stage specific TRAP (Figure S10d).

Comparing between AM-stage specific lines, the number of enriched/depleted genes were in line with the expected timeframes for promoter activity (*pAM1* having the broadest range and *pARK1* the narrowest). *pAM1:TRAP* exhibited the greatest number of enriched transcripts (1,588 up/4041 down), followed by *pPT11:TRAP* (1,652 up/1,957 down) and *pARK1:TRAP* (1,402 up/106 down), consistent with these lines allowing to capture distinct cell-states or populations through AM development (Fig. 5c). Overlaps between these gene lists also followed the expected developmental trajectories of these cell populations. *pAM1:TRAP* and *pPT11:TRAP* shared the largest proportion (34.7%), while *pAM1:TRAP* and *pARK1:TRAP* shared the smallest (9.7%). *pPT11:TRAP* was mostly recapitulated by the other 2 lines, while *pAM1* and *pARK1* had substantial unique signatures. For *pAM1*, the majority of these were depleted, conversely for *pARK1* the majority were enriched, thus providing a unique and much-needed resource to investigate transcriptional reprogramming during early symbiotic interaction and arbuscule collapse (Fig. 5d, Tables S6, S7).

To further validate the capture of distinct cell-populations by AM-stage specific TRAP, we examined the abundance patterns of a set of known cell-identity markers identified from the literature, including single-cell and spatial transcriptomic datasets in rice (Liu et al. 2021; Wang et al. 2021; Zhang et al. 2021; Zhu et al. 2025) (Table S8). The vast majority of cell-type specific genes were differentially regulated in the AM-stage specific TRAP profiles, in contrast to ubiquitous controls such as *OsCc1* and to the total mRNA +Ri vs −Ri comparison (Fig. 5e, Figure S11). Most markers were reduced in *pAM1* and *pPT11* TRAP relative to total mRNA or *p35S*:TRAP, particularly those associated with vascular cell types. This is consistent with TRAP capturing colonized cortical cells, with stele-specific transcripts being underrepresented. Interestingly, this trend is absent in *pARK1* TRAP comparisons. Instead, several cortical, exodermal, epidermal and endodermal markers appeared enriched both in *pARK1* and *pPT11*, but depleted in *pAM1*. These dynamic profiles cannot be explained by capture of cortical or epidermal cell types alone. Together with results from Molecular Cartography (Figure S12), this suggests dynamic repatterning of cell-identity markers across AM development, with initial downregulation during early colonization and arbuscule formation, followed by recovery during arbuscule collapse. Notably, many cell-type markers were also differentially represented in *p35S* TRAP and total mRNA pools, suggesting either translational regulation of cell-identity markers, or incomplete capture of all cell types by *p35S:TRAP.* Finally, even within ubiquitous control genes, cytoskeletal components such as actin (*OsACT2*) and tubulin (*OsTUB1*) were significantly reduced in AM-stage specific TRAP profiles, especially *pPT11*, consistent with the extensive cytological remodeling associated with arbuscule development (Fig. 5e, Figure S10).

### Temporal dynamics of gene activity across AM development

**Figure 6:**
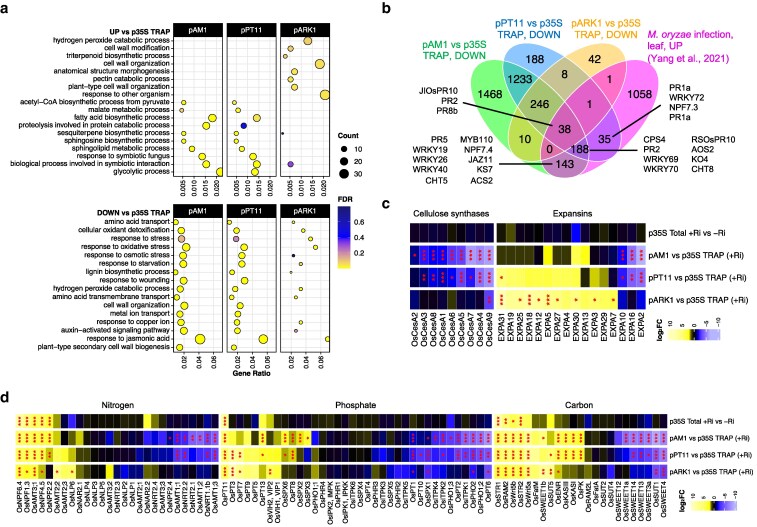
Dynamic regulation of biological processes, including defence, cell-wall biosynthesis and nutrient signaling, at specific stages of AM development. a) GO term enrichment plot for up and downregulated genes in the translatomes obtained by use of 3 AM-stage specific TRAP lines compared with the *p35S:TRAP* line. Top 10 GO terms by gene ratio (proportion of genes in dataset out of all known genes in the GO term) for each set are shown; color scale indicates significance as false discovery rate (FDR); dot size proportional to the number of genes in each set included in the GO term. b) Venn diagram showing overlaps between downregulated genes for *pAM1*, *pPT11* and *pARK1* vs *p35S*:TRAP samples, compared with activated genes in *M. oryzae* infected leaves (Yang et al. 2021). c) Heatmap of log_2_ fold-changes (log_2_FC) for a selection of comparisons of interest, for cellulose synthases and expansins (full comparison list on Figure S14b). d) Heatmap of log_2_FC for a selection of comparisons of interest (full comparison list on Figure S15), for key genes related to phosphate, nitrogen and carbon transport, perception and signaling. Significance levels as determined by DESeq2 shown with asterisks (* for *P*-value <0.05, ** for <0.01, *** for <0.001). Genes were subjected to hierarchical clustering within each group.

To identify biological processes dynamically regulated during symbiosis, we performed Gene Ontology (GO) term enrichment analysis on differentially captured genes from each AM-stage specific *TRAP* line compared with *p35S*. This revealed stage-specific functional programs consistent with promoter activity. In *pAM1:TRAP* and *pPT11:TRAP*, enriched genes corresponded to AM-related processes such as “response to symbiotic fungus” and “lipid metabolism,” while *pARK1:TRAP* instead highlighted “cell wall organization,” “pectin catabolism,” and “hydrogen peroxide catabolism” (Fig. 6a). This differential enrichment for *pARK1:TRAP* suggests substantial functional alterations during late stages of arbuscule development. Depleted genes in *pAM1:TRAP* and *pPT11:TRAP* included components of the strigolactone signaling pathway (*OsD14*, *OsSXMLs*) as well as biosynthetic enzymes (*OsMAX4/DAD1*, *OsCXE15s*), reflecting a potential down-regulation of strigolactone biosynthesis and signaling in colonized tissue (Figure S13). However, strigolactone biosynthetic enzymes *OsD17/CCD7* and *OsD10/CCD8B* appear enriched in *pAM1* and *pPT11:TRAP*, pointing to more nuanced metabolic alterations in the arbusculated cell. Also decreased were transcripts of genes linked to stress and immunity, such as responses to jasmonic acid (JA) and oxidative stress. Conversely, stress-related terms (eg “hydrogen peroxide catabolism,” “response to other organism”) were enriched among *pARK1:TRAP* upregulated genes. Comparing depleted genes with those induced by pathogenic *Magnaporthe oryzae* infection (Yang et al. 2021) showed broad overlap for *pAM1:TRAP* and *pPT11:TRAP* but limited overlap for *pARK1:TRAP* (Fig. 6b). These included classical defense genes (encoding PR proteins, WRKYs, chitinases) and associated pathways (JA and ethylene signaling, flavonoid biosynthesis, redox regulation), supporting the idea that AM development fine-tunes immunity; downregulated during colonization and maintenance, but reactivated during arbuscule collapse (Figure S14a, Tang et al. 2021). Cell wall-related terms were similarly highly enriched in a stage-dependent manner. *pAM1:TRAP* and *pPT11:TRAP* showed depletion of cellulose synthases and expansins, while the latter were enriched in *pARK1:TRAP*, consistent with the requirement for wall loosening during arbuscule formation and remodeling during degeneration (Fig 6a, c, Figure S14b).

We further investigated stage-specific profiles by identifying genes uniquely enriched or depleted under a particular promoter line. Among *pAM1:TRAP*-unique depleted genes (1,611 in total), at least 138 encoded predicted transcription factors suitable as candidates with potential regulatory roles. In addition, at least 138 genes had predicted protein kinase activity, consistent with suppression of defense signaling cascades that might otherwise block colonization (Table S9). In *pARK1:TRAP*, uniquely enriched genes (1,713 in total) included 185 genes involved in response to stimulus including 19 peroxidases, and at least 60 genes with predicted hydrolase activity (Table S10). Four ribosomal small subunit and 6 ribosomal large subunit genes were also identified. Included were 2 *RPL23s*, a gene family known to be highly expressed under various abiotic stresses (Moin et al. 2016; Saha et al. 2017) and implicated in arbuscule collapse in *Medicago* (Floss et al. 2017). We also identified at least 59 genes encoding proteins with predicted TF activity, including an orthologue of the master regulator and maize-domestication gene *Teosinte-branched 1* (*Tb1*), named *OsTb2* or *OsREP1* (Lyu et al. 2020). These represent candidate genes regulating the post-arbuscule development stage (Table S10).

Nutrient transporters and signaling components also showed interesting patterns. Beyond the induction of *OsPT11* and *OsPT13*, TRAP-seq revealed complex reprogramming of non-symbiotic phosphate transporters and signaling regulators undetectable in total mRNA profiles: *OsPT8* and *OsSPX2/6* were enriched in AM-stage specific lines, whereas *OsPT1/2/6* and *OsPHO1;2/2* were depleted. Activation of *OsPT3* and *OsPT7* in *pPT11:TRAP* and *pARK1:TRAP*, and of *OsPT8* in *pAM1:TRAP* and *pPT11:TRAP*, further highlighted developmental resolution of phosphate signaling (Fig. 6d, Figure S15). Similarly, nitrogen and carbon transporters (*OsAMTs, NRTs, SWEETs, SUTs*) displayed complex patterns of activation or repression in an AM-stage-specific manner. Many of these AM-regulated transcripts were not differentially abundant in bulk RNA-seq, particularly for genes repressed in early (*pAM1*) and mature (*pPT11*) stages. Comparison with our spatial transcriptomics dataset confirmed most nutrient-related trends, including depletion of *OsSWEET1a, OsSWEET14, OsPT6, OsPHO1;2, OsPHO2* and *OsSPX1*, which again were not detected as downregulated in bulk RNA-seq (Figure S12). However, spatial transcriptomics also revealed changes in spatial distribution, such as *OsSWEET12*, that were not associated with detectable alterations in abundance at the transcript or translated mRNA levels (Figs. 2d, 6d, Figure S12). Together, these complementary approaches demonstrate that AM symbiosis involves fine-scale, dynamic modulation of immunity, cell-wall remodeling, hormone signaling, and nutrient transport, with TRAP-seq providing developmental and translational resolution not captured by bulk transcriptomics.

## Discussion

By integrating spatial transcriptomics with AM-stage specific TRAP-seq, this study provides a high-resolution, cell-type-specific view of mRNA abundance and dynamics during symbiosis, offering insights into the spatial and temporal coordination that underlies AM development.

Our spatial analysis surprisingly revealed transcript abundance for *RiEF1a*, an essential fungal translational elongation factor, was higher in vesicles and hyphae than in arbuscules, despite the latter's central role in nutrient exchange. This discrepancy may be partially explained by differences in nuclear distribution or by the more pronounced translational activity in vesicles. Vesicles and hyphae harbor visibly higher densities of fungal nuclei, likely supporting ongoing metabolic activity and cellular growth. In contrast, arbuscules, particularly their fine terminal branches, are structurally constrained, which may physically limit nuclear entry (Bianciotto et al. 1995). Rather than reflecting a reduced need for transcription, lower nuclear density in arbuscules may necessitate transcript or protein movement from the trunk and connected, adjacent fungal hyphae. This model is supported by previous knowledge from fungal species such as *Ustilago maydis*, where transcripts and proteins are actively transported over long distances within multinucleate hyphal networks (Zarnack and Feldbrügge 2007, 2010). Therefore, vesicles and hyphae may serve as biosynthetic hubs that support the specialized functions of arbuscules.

Our analysis also uncovered reduced transcript abundance for most cell-identity markers in roots undergoing AM colonization. This was further validated using TRAP-seq, where cell-identity markers showed significant alterations in their abundance in distinct cell-populations during AM symbiosis, particularly downregulated in early and middle stages, while recovered during arbuscule collapse. These results both emphasize the need for systematic characterization of cell-type markers under abiotic and biotic stresses, in distinct root zones and cell types; as well as highlight that deep transcriptional and translated mRNA changes occur in AM-colonized tissue, even regarding cell-identity.

However, our spatial transcriptomic analysis failed to accurately predict highly dynamic AM developmental stages, particularly for the arbuscule-containing cell. We found a striking transcriptional heterogeneity among arbuscules, which only in part could be attributed to the dynamic transcriptional regulation across their development, as this was observed between arbuscules at the same developmental stage. Even within the same tissue, morphologically equivalent arbuscules displayed marked differences in expression of key arbuscule localized genes such as *OsPT11*, *OsNPF4.5*, *OsSTR1*, and *OsRAM2*. Through single-cell transcriptomic analyses we identified distinct subclusters of arbusculated cells, differentiating “*canonical*” (expressing known arbuscule-specific genes) and “*non-canonical*” (conversely enriched in other transcripts such as *OsSWEET12* and *OsPT8*) transcriptional signatures, although substantial cell-to-cell variability was still evident. This suggests either highly dynamic transcriptional changes within narrow developmental windows, or that arbuscule function is “cell-autonomous,” fine-tuned in response to local physiological or developmental cues. These findings challenge the concept of a uniform “mature” arbuscule, and highlight the need to investigate arbuscule cellular heterogeneity, its regulatory causes, and functional consequences for nutrient transport.

AM-stage specific TRAP-seq complemented spatial transcriptomics, providing developmental resolution by capturing ribosome-associated transcripts in colonized cell populations at distinct progressive stages of the interaction. Furthermore, while spatial transcriptomics revealed where transcripts accumulate within the cell, TRAP-seq provided a complementary perspective by identifying transcripts that had been recruited for translation. This approach uncovered extensive and dynamic translational reprogramming, particularly involving immunity, cell-wall and stress-related genes, including JA signaling components, WRKY transcription factors, cellulose synthases and expansins, which were generally downregulated during early stages of colonization, and upregulated in later stages. Our results thus shed light to the role of these biological processes in AM symbiosis, which were historically cause of debate due to their complexity, particularly in the case of JA signaling (Gutjahr et al. 2015), immunity (García-Garrido and Ocampo 2002) and cell-wall related genes (Balestrini and Bonfante 2014). Our findings suggest these processes are dynamically regulated across AM development, initially suppressed to enable fungal entry and accommodation, then re-activated during arbuscule degradation.

Beyond immune modulation, our TRAP-seq data revealed dynamic regulation of genes involved in nutrient transport and signaling during arbuscule maintenance. As expected, symbiotic phosphate transporters such as *OsPT11* and *OsPT13* were enriched at the transcript and translated mRNA levels. However, AM-stage specific TRAP-seq identified uncharacterized modulation of direct-uptake phosphate transporters and signaling components, both induced (*OsPT3*, *OsPT7*, *OsPT8, OsSPX2, OsSPX6*) and repressed (*OsPT1*, *OsPT2*, *OsPT6; OsPHO1;2; OsPHO2*), in some cases dynamically through AM development, reflecting a stage-specific and nuanced control of phosphate uptake and signaling. Similar regulation, also undetectable in bulk RNA-seq, was observed for nitrogen and carbon transporters, including *OsAMTs*, *OsNRTs*, *OsSWEETs*, and *OsSUTs*. These findings highlight the pivotal role of local dynamic control in fine-tuning host nutrient management throughout the symbiotic timeline.

During later stages of AM fungal development, we observed a coordinated upregulation of immunity and stress-related genes, indicative of arbuscule degeneration. This transition echoes findings in *Medicago truncatula*, where arbuscule collapse was associated with reactivation of defense pathways and cellular recycling processes (Floss et al. 2017). Importantly, TRAP-seq uncovered candidate genes potentially involved in late stages of arbuscule development and collapse. For instance, the large ribosomal subunit gene *OsRPL23* showed marked enrichment in late-stage colonized cells and shares homology with a Medicago gene previously implicated in arbuscule turnover (Floss et al. 2017). In addition, *OsREP1/OsTB2*, orthologue of maize-domestication gene *Tb1* encoding a TCP-family transcription factor, was one of the most highly enriched genes during the post-arbuscule development stage, suggesting a potential regulatory role in fungal withdrawal or reactivation of host developmental programs.

Integrating TRAP-seq with spatial transcriptomics provided complementary insights beyond what either approach reveals alone. The datasets cross-validated patterns of gene enrichment and depletion, particularly for genes not detected as differentially expressed in bulk RNA-seq (such as cell-type markers, AM signaling components *OsD14L, OsCEBiP*, *OsCERK2*, and nutrient transporters *OsSWEET1a*, *OsPT6*, *OsPHO1;2*, *OsPHO2*; Figure S12). Moreover, TRAP-seq added temporal resolution to spatial patterns: for instance, cell-type marker genes depleted in colonized regions show developmental dynamics, being repressed during early and mature arbuscule stages but reactivated during senescence. Conversely, spatial transcriptomics revealed localized expression heterogeneity masked in TRAP-seq, exemplified by *OsSWEET12*, which shows arbuscule-specific depletion despite not being differentially captured in TRAP or bulk RNA-seq. This integration thus contextualizes translatome changes with subcellular spatial resolution while providing developmental dynamics to spatial snapshots.

Together, our complementary approaches provide distinct but intersecting views of AM symbiosis. While the microbe programmed cortical cell translatome reveals actively translated genes within defined cell populations, spatial transcriptomics reveals expression landscapes within the broader tissue context. Integrating these perspectives reshapes our understanding of gene regulation during arbuscule development, function, and turnover, revealing a complex, multilayered regulatory network. Ultimately, our findings redefine key aspects of AM symbiosis by revealing transcriptional and translational heterogeneity across fungal structures and host cells, identifying immune reprogramming as a central feature of early accommodation, and uncovering finely tuned spatial regulation of nutrient transporters. Moving forward, combining these insights with proteomic analyses, live-cell imaging, and targeted genetic studies will be essential for mapping protein localization, validating candidate regulators, and decoding the molecular dialogue underpinning arbuscule development.

## Materials and methods

### Plant growth and inoculation

Seeds of *Oryza sativa cv. Japonica*, Nipponbare were surface-sterilized briefly in 70% (v/v) ethanol, then for 20 min in 3% (v/v) sodium hypochlorite. Imbibed seeds were germinated on 0.9% (w/v) bactoagar at 30 °C for 7 d. Pre-germinated seedlings were transferred into pots (11 × 8 cm) containing sterile quartz sand in walk-in growth chambers at 12-h:12-h, light:dark cycle at 28:20 °C and 60% relative humidity. Plants were inoculated with 900 spores of *R. irregularis* DAOM197198 (Mycorise ASP, Premier Tech Biotechnologies, Rivière-du-loup, Canada). Plants were watered 3 times weekly for the ﬁrst 2 wks post-inoculation (wpi), thereafter fertilized twice a week with half Hoagland solution (25 µM Pi) and 0.01% (*w/v*) Sequestren Rapid (Syngenta), as previously described by Gutjahr et al. (2008). Plants required for spatial transcriptomics experiments were harvested at 6 wpi.

For TRAP-seq experiments, 6 wpi wild-type plants were retained as “nurse plants” to enable rapid colonization of young test plants. Here, 5 pre-germinated seedlings were sown into pots, each already containing a colonized nurse plant. Test plants were harvested at 3 wpi.

### Molecular Cartography

#### Molecular Cartography probe design

Probes were designed using Resolve BioSciences' proprietary design algorithm and gene annotations from the *Oryza sativa* ssp. *japonica* Nipponbare reference genome (Os-Nipponbare-Reference-IRGSP-1.0.58) (Kawahara et al. 2013). Searches were confined to the coding regions to identify potential off-target sites. Each target sequence underwent a single scan for all *k*-mers, favoring regions with rare k-mers as seeds for full probe design. A probe candidate was generated by extending a seed sequence until reaching a certain target stability. After these initial screens, probes were aligned with the background transcriptome, and probes with stable off-target hits were discarded. From the pool of accepted probes, the final set was composed by selecting the highest scoring probes.

#### Identification and preparation of AM-colonized root tissue

To facilitate the identification of AM-colonized root regions, we used a *pSCAMP:eGFP-SCAMP* rice reporter line expressing a GFP-tagged secretory carrier membrane protein, generated by Kobae and Fujiwara (2014). Roots were harvested at 6 wpi and screened for eGFP fluorescence using a Leica M205 FA stereomicroscope. GFP-positive root segments (0.3 to 0.5 cm in length) were immediately processed for spatial transcriptomics.

#### Tissue preparation and molecular cartography

Root segments were fixed in paraformaldehyde and embedded in paraffin according to Resolve Biosciences' standard plant tissue preparation protocol, adapted from Zöllner et al. (2021). Semi-thin sections (10 µm) were made using a Leica HistoCore Autocut R microtome and adhered to the Molecular Cartography slides. Sections underwent deparaffinization, permeabilization, and refixation according to Resolve BioSciences' user guide. Samples were mounted in SlowFade Diamond Antifade Mountant (Thermo Fisher Scientific) and shipped to Resolve Biosciences for Molecular Cartography analysis.

At Resolve BioSciences, sections were washed twice in 1× phosphate buffered saline for 2 min, followed by 1 min washing in 50% and 70% ethanol at room temperature. Ethanol was removed by aspiration and DST1 buffer was added, followed by tissue priming for 30 min at 37 °C and by a 48 h hybridization using probes specific for the target genes. Samples were washed to remove excess probes and fluorescently tagged in a 2-step color development process. Fluorescent signals were removed after imaging in a decolorization step. Colorization, imaging, and decolorization were iterated for multiple cycles to generate a unique combinatorial code for each target gene. Samples were imaged by Resolve BioSciences using a Zeiss Celldiscoverer 7 with a 50× Plan Apochromat water immersion objective having a numerical aperture (NA) of 1.2 and a 0.5× magnification changer, resulting in a final magnification of 25× (Glenn et al. 2023). To visualize fungal structures, sections from experiment 1 were stained with WGA-AF633 (Invitrogen), although staining was suboptimal. In experiment 2, WGA-AF488 was used to improve fungal structure visibility. All sections were counterstained with DAPI to visualize both plant and fungal nuclei. Probes yielding fewer than 20 transcript counts per section across both −Ri and +Ri conditions were excluded from downstream analyses (detailed in Table S1). Additionally, root sections with significant tissue damage or low overall transcript abundance were omitted from further analysis (detailed in Table S2).

#### Image and transcript analysis

Image analyses, including generation of image-transcript overlays and manual cell-boundary segmentation was performed in Fiji under ImageJ software license (Schindelin et al. 2012). Transcript abundances in vesicles and arbuscules were determined by manually segmenting vesicles and arbusculated cells in inoculated (+Ri) sections, extracting transcript spot data for all genes in the probe set for each individual structure, as well as its area, then calculating the transcript abundance per unit area (μm^2^) for each gene in each structure, comparing arbuscules versus vesicles. Transcript CL analysis was carried out using the Polylux plugin (Resolve Biosciences). Default settings were used for transcript CL analyses (*xy*-step size = 1, *z*-step size = 3.5, search radius = 50, and distance penalty = 4). The algorithm calculates CL scores by searching within a defined neighborhood around each transcript at pixel resolution and scoring the frequency of finding specific transcript pairs, with non-linear distance weighting applied. To account for section-to-section variability, we calculated the median transcript CL score separately for all transcripts within mock and mycorrhizal samples. Single-cell clustering analysis was performed using the Seurat package (v5.3.1) in R. Low-quality cells were filtered using the criteria nFeature_RNA > 3 and nCount_RNA < 2,000, resulting in 2,810 retained cells. Data were normalized (LogNormalize, scale factor 10,000), and highly variable features were identified (2,000 features, vst method). After scaling and PCA (10 dimensions), cells were clustered using the Louvain algorithm at resolution 0.5, yielding 6 clusters. UMAP was applied for visualization. Cluster identities were mapped back to their original spatial context using ImageJ with the Polylux plugin (Resolve Biosciences).

#### Translating ribosome affinity purification (TRAP) and RNA-Seq

*Promoter:TRAP* constructs were prepared with the TRAP destination vector *pH7WG-OsTRAP* which was engineered by modifying *p35S:HF-OsRPL18* (Reynoso et al. 2022). This vector contains a Gateway recombination site for promoter insertion upstream of a chimeric ribosomal protein fusion comprising a 6×His-FLAG-3×Gly tag, GFP, and OsRPL18/eL18 (*Os03g0341100*). Promoters listed in Table S15 were cloned into pENTR-D/TOPO (Invitrogen) and subsequently recombined into the TRAP vector via LR Clonase II enzyme mix (Invitrogen). All constructs were validated by Sanger sequencing. Resulting T-DNA plasmids were introduced into *Oryza sativa japonica* cv. Nipponbare embryogenic calli, derived from mature seed embryos, through *Agrobacterium tumefaciens-*mediated transformation. Callus for transformation of rice cultivar Nipponbare was generated by plating surface-sterilized mature seed, with embryo axes removed, on N6DT medium (3.95 g/L N6 basal salts, 30 g/L sucrose, 300 mg/L casein hydrolysate, 100 mg/L myo-inositol, 2,878 mg/L proline, 0.5 mg/L nicotinic acid, 0.5 mg/L pyridoxine HCl, 1 mg/L thiamine HCl, 37.3 mg/L Na_2_EDTA, 27.8 mg/L FeSO_4_, 2 mg/L 2,4-D Na salt, 150 mg/L Timentin, 4 g/L Gelrite, pH 5.8). Plates were sealed with Parafilm and cultured in the dark at 28 °C for 21 d after which time callus was cut into 2 to 4 mm pieces, plated on fresh N6DT and cultured as before for a further 4 d. Preparation of Agrobacterium strain EHA105 containing the binary constructs and transformation of the rice callus pieces was carried out as previously described (Choi et al. 2020). T2 generation plants were used for TRAP experiments.

TRAP was performed as previously described (Mustroph et al. 2009a; Reynoso et al. 2015) with modifications outlined by Reynoso et al. (2019). From each sample, both total RNA and TRAP RNA (following ribosome purification) were extracted using the RNeasy Micro Kit (Qiagen). Libraries were prepared with the QuantSeq 3′ mRNA-seq kit (Lexogen) incorporating unique dual indices (UDIs) and unique molecular identifiers (UMIs) for 3 biological replicates per treatment. Sequencing was performed on an Illumina NovaSeq 6000 platform generating 150 bp single-end reads. Sequencing data was analyzed by the Lexogen NGS Kangaroo Data Analysis Platform including quality control, trimming, UMI deduplication, STAR alignment to the *O. sativa* Nipponbare reference transcriptome (Os-Nipponbare-Reference-IRGSP-1.0.58) (Kawahara et al. 2013) and count quantification. To address low read counts in AM-stage specific TRAP samples, an additional library was constructed and sequenced from the original RNA extractions, with resulting counts combined at the count level. Subsequent data analysis, including normalization, filtering and differential gene expression, was carried out in R using the DESeq2 v1.40.2 package (Love et al. 2014), with a threshold of fold-change ≥1.5 or ≤−1.5 and Benjamini–Hochberg false discovery rate corrected *P*-value <0.05. GO enrichment analyses were conducted using the R package clusterProfiler v4.8.3 (Wu et al. 2021). The annotations of the genes including associated GO terms were collected from various sources: The Rice Annotation Project (Kawahara et al. 2013) and EnsemblPlants (Martin et al. 2023) annotations for the Os-Nipponbare-Reference-IRGSP-1.0.58 reference genome and Oryzabase (Kurata and Yamazaki 2006).

### Accession numbers

All accession numbers for *Oryza sativa* and *Rhizophagus irregularis* genes and proteins are detailed in the supplemental information: Table S1 for Molecular Cartography targets, and Tables S6 to S14 for genes mentioned in the TRAP-seq section of the results.

## Supplementary Material

koag133_Supplementary_Data

## Data Availability

Raw sequences, images and gene count matrices generated in this study are available in the NCBI GEO database under the accessions GSE308140 (for total and TRAP mRNA sequencing) and GSE316219 (for Molecular Cartography data). R code employed to analyze the data and generate graphics is available in the following github repository: https://github.com/gabriel-ferreras/Spatial_rice_AMF_2025. Other data supporting the findings of this article are available in the supplemental information.
