# Supplementary material for: Spatiotemporal regulation of arbuscular mycorrhizal symbiosis at cellular resolution
Source: Plant Cell. 2026 May 11;38(6):koag133. doi: 10.1093/plcell/koag133 (PMC13237562; doi:10.1093/plcell/koag133)
Supplement: koag133_Supplementary_Data [file koag133_supplementary_data.zip › Chancellor_et_al_figuresupp_edited.pdf]

Spatiotemporal regulation of arbuscular mycorrhizal symbiosis at cellular resolution

Tania Chancellor and Gabriel Ferreras-Garrucho, Garo Z. Akmakjian, Héctor Montero, Sarah Bowden, Matthew S. Hope, Emma Wallington, Samik Bhattacharya, Christian Korfhage, Julia Bailey-Serres, Uta Paszkowski

Supplementary data

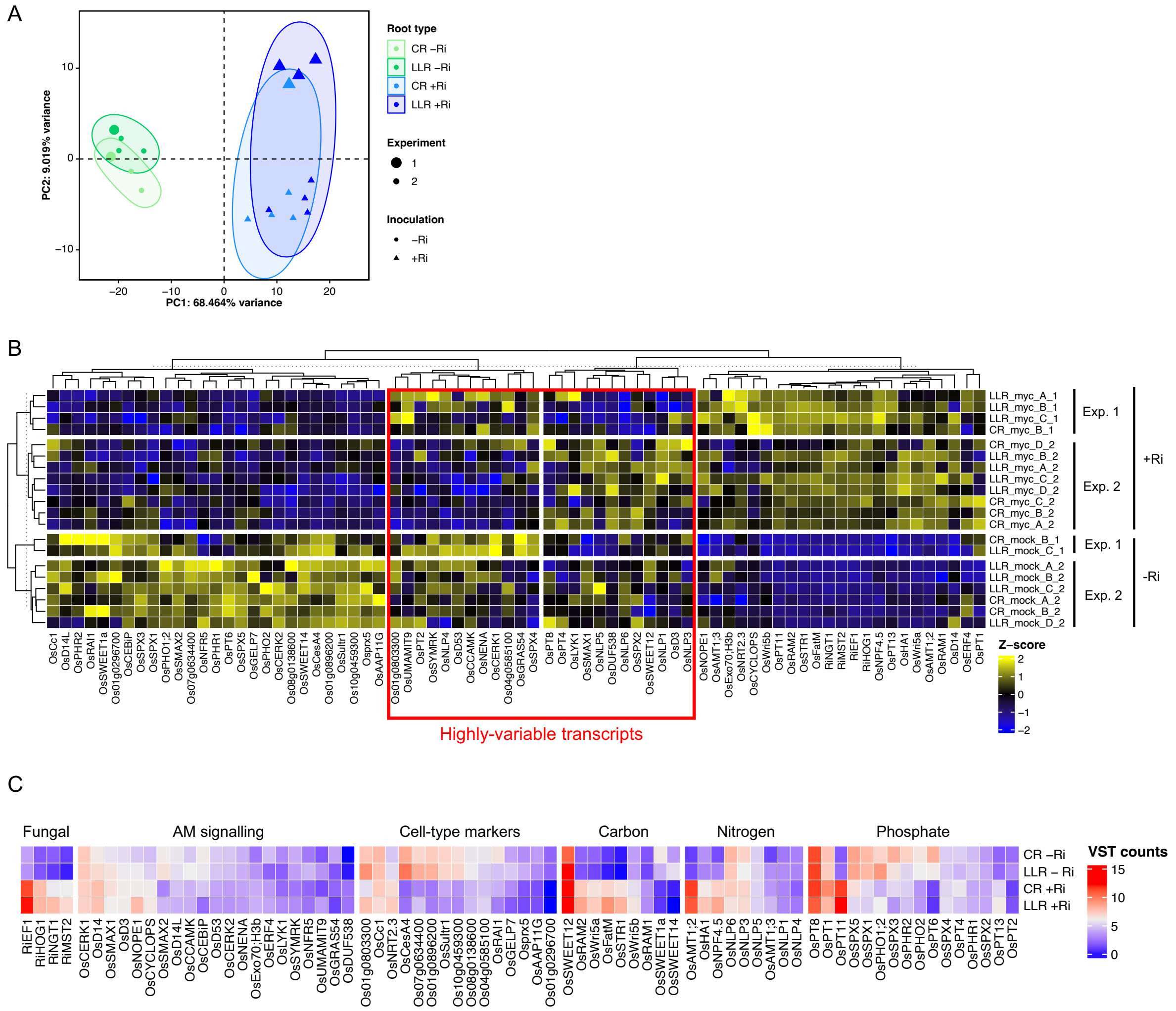

Supplementary Figure S1. Validation of Molecular Cartography results. Supports figure 2. (A) Principal Components Analysis (PCA) for spatial transcriptomic sections, separated based on root type (colour, light for CR and dark for LLR), experiment (size) and inoculation (shape, circles for -Ri, triangles for +Ri). Variance-stabilised counts (VST counts) for transcript spot data for all filtered probes for each section were used to conduct PCA analysis. Only PC1 and PC2 are shown as they together account for more than 50% of the variance. Best fit ellipses for each sample type were created using the Khachiyan algorithm from the *ggforce* package in R (Pedersen, 2025). (B) Heatmap of VST row-centered counts for transcript spot data of each section. Red box highlights 24 low-abundance transcripts that are differentially captured between experiments, causing

apparent batch effect between experiments. Genes and sections were subjected to hierarchical clustering followed by k-means partitioning to subdivide the set in four groups. CR, crown root; LLR, large lateral root; Ri, *Rhizophagus irregularis* inoculation. (C) Heatmap of VST counts for averaged transcript spot data for all sections of each condition (root type x inoculation). CR, crown root; LLR, large lateral root; Ri, *Rhizophagus irregularis* inoculation.

A

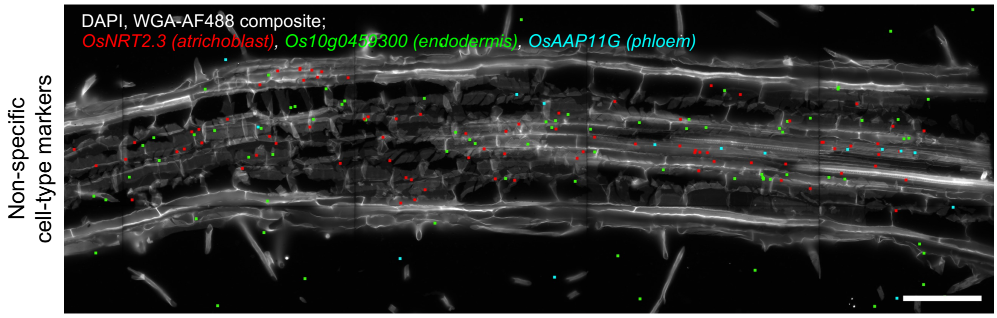

B

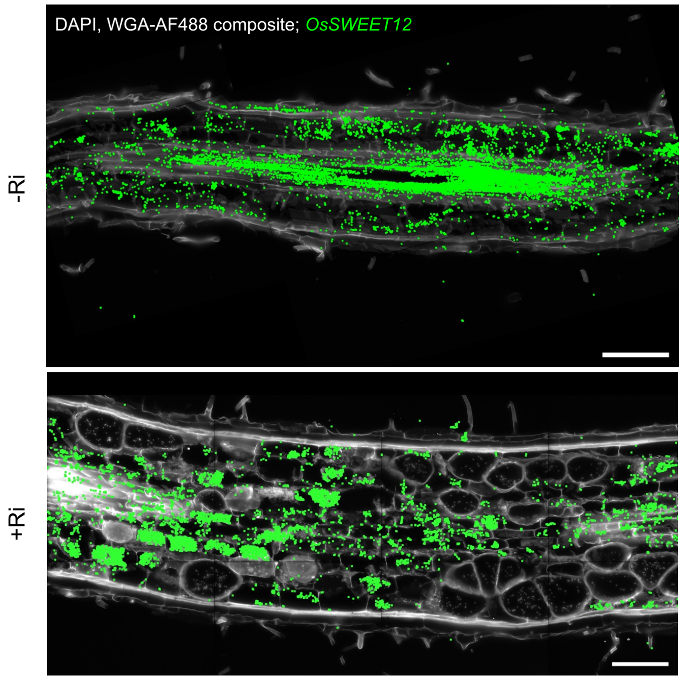

Supplementary Figure S2. Non-specific cell-type marker genes and replicate of *OsSWEET12* redistribution upon AM colonisation. Supports figure 2. (A) Image-transcript overlay image of a non-inoculated (-Ri) section showing spatial expression of a selection of cell-type markers that were not specific to the predicted cell-type in our samples. (B) Image-transcript overlays highlighting spatial patterning of *OsSWEET12* in alternative -Ri and +Ri sections. DAPI/WGA-AF488 composite in white, placed on a black background, different colours correspond to independent transcripts, each spot corresponds to one detected transcript. Dotted square and arrows highlight transcript abundance heterogeneity between arbuscules at the same developmental stage. Scale bar, 100 µm.

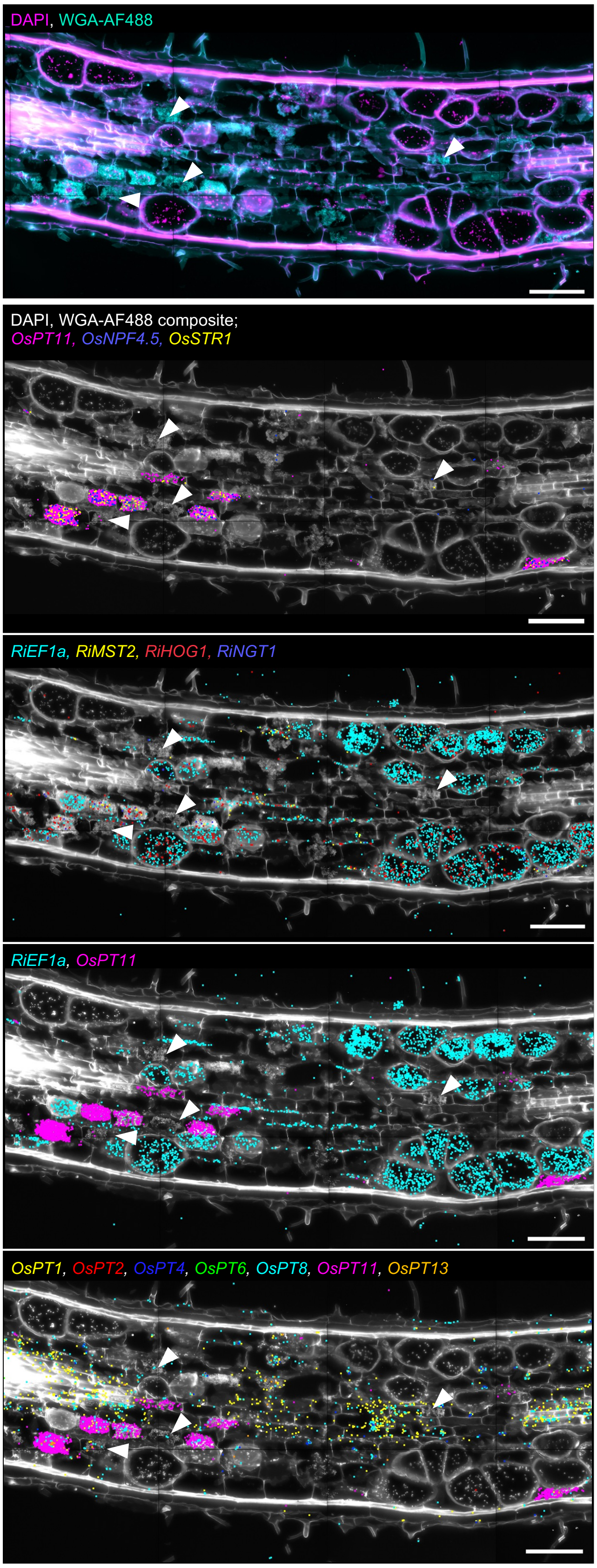

Supplementary Figure S3. Molecular Cartography results for an inoculated (+Ri) section of a crown root (CR). Supports figure 3. First panel shows composite image of DAPI (magenta) and WGA-AF488 (cyan) for visualization of nuclei, cell boundaries and fungal structures. Rest of panels

show image-transcript overlays for a selection of plant genes, fungal genes, one fungal and one plant gene, and plant phosphate transporters, in that order. DAPI/WGA-AF488 composite in white, placed on a black background, different colours correspond to independent transcripts, each spot corresponds to one detected transcript. Scale bar, 100 µm.

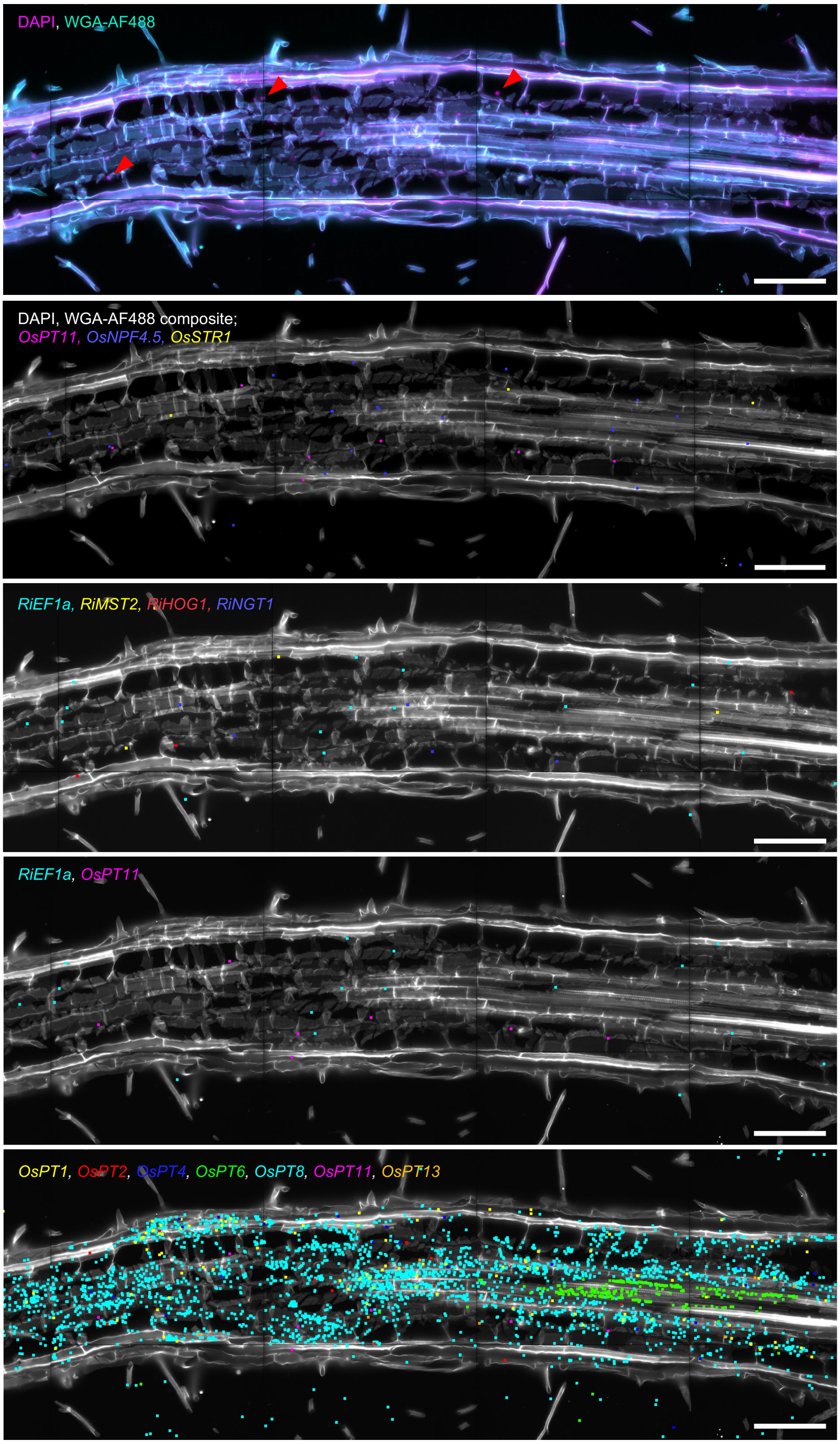

Supplementary Figure S4. Molecular Cartography results for a non-inoculated (-Ri) section. Supports figure 3. First panel shows composite image of DAPI (magenta) and WGA-AF488 (cyan) for visualization of nuclei and cell boundaries. Rest of panels show image-transcript overlays for a selection of plant genes, fungal genes, one fungal and one plant gene, and plant phosphate

transporters, in that order. DAPI/WGA-AF488 composite in white, placed on a black background, different colours correspond to independent transcripts, each spot corresponds to one detected transcript. Scale bar, 100 µm.

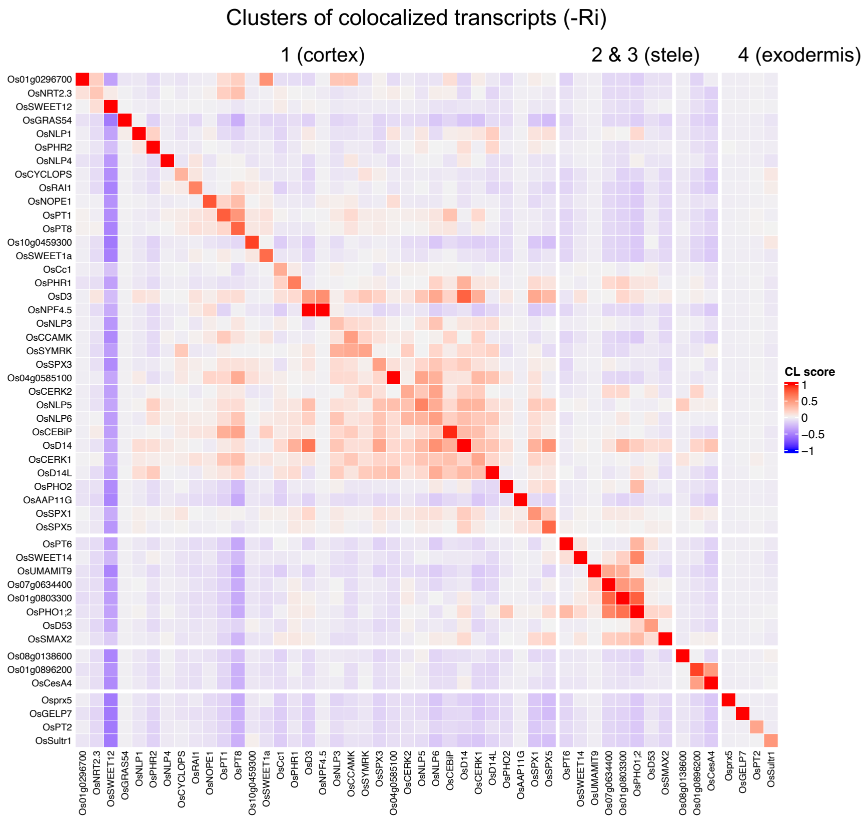

Supplementary Figure S5. Colocalization heatmap of transcript colocalization scores for non-inoculated (-Ri) sections. Supports figure 4. Genes with >0.2 self-co-localisation scores were used for analysis, subjected to hierarchical clustering, distinct clusters indicated by thicker boundaries between genes, as well as on top, with the associated cell-type with each cluster in brackets. Colour scale represents the transcript colocalization (CL) score.

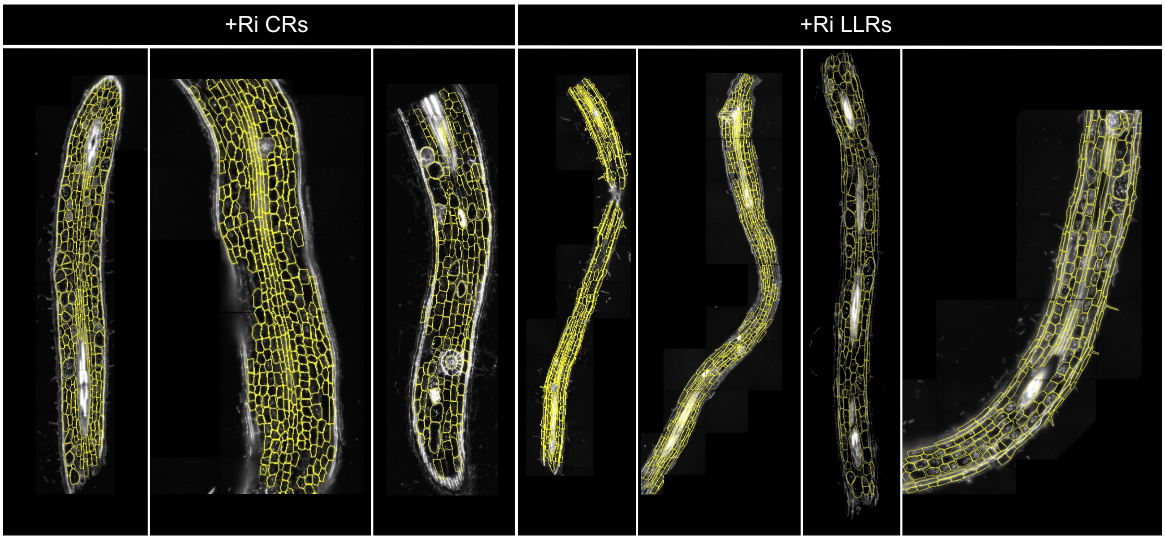

Supplementary Figure S6. Cell-segmentation of Molecular Cartography +Ri sections. Supports figure 4. Yellow lines indicate segmented cells, DAPI/WGA-AF488 composite in white, placed on a black background. Separated crown roots (CR) and large lateral roots (LLR).

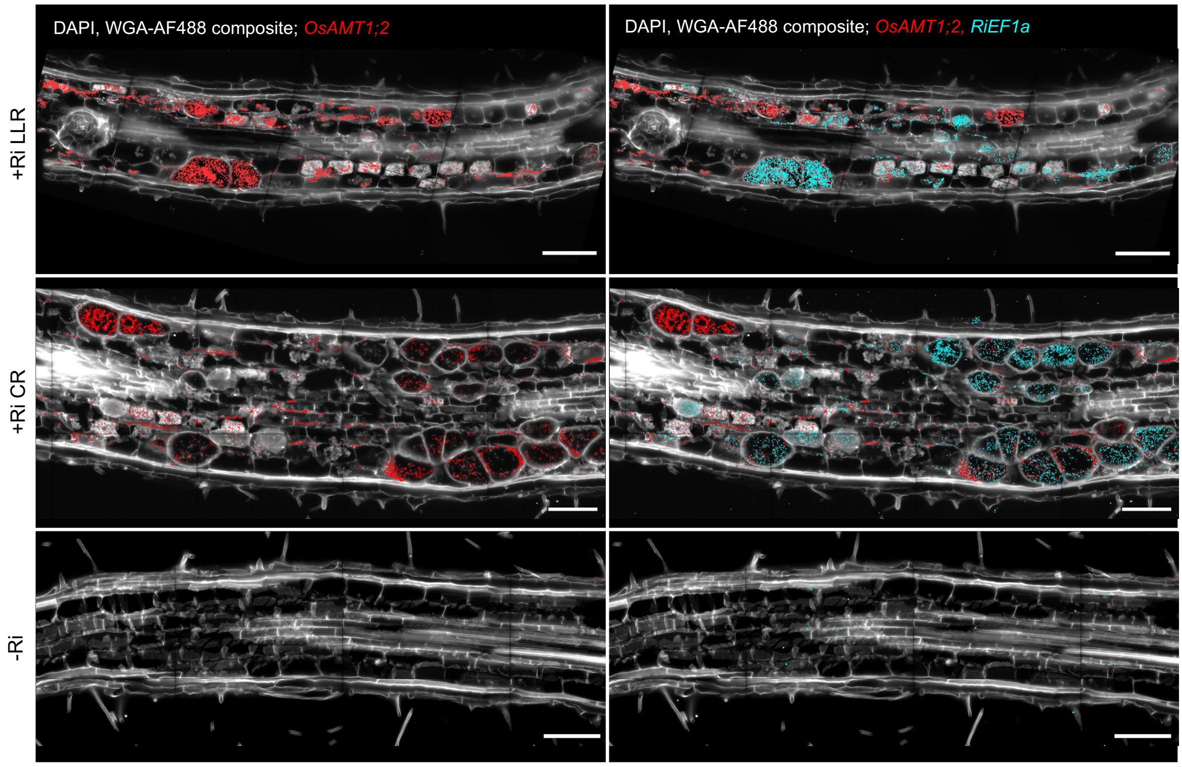

Supplementary Figure S7. Distribution of *OsAMT1;2* transcripts in mycorrhizal roots. Supports figure 4. Image-transcript overlays highlighting spatial patterning of *OsAMT1;2* in +Ri and -Ri sections. DAPI/WGA-AF488 composite in white, placed on a black background, each red spot corresponds to one detected transcript. Scale bar, 100 µm.

A

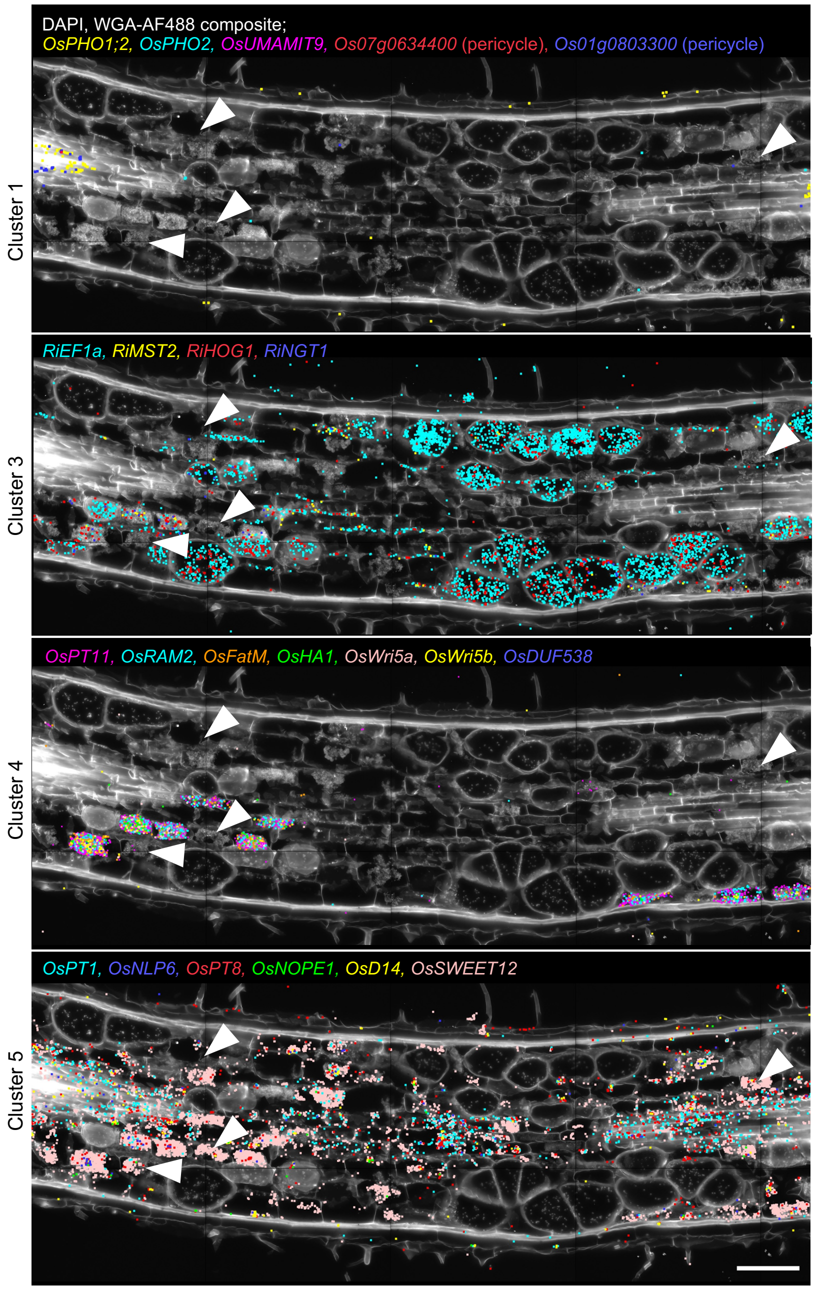

B

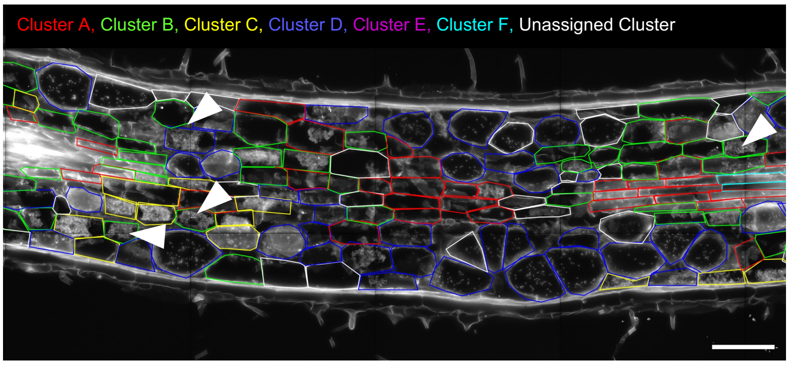

Supplementary Figure S8. Co-localisation clusters and cell-segmentation clusters for an alternative inoculated (+Ri) crown root (CR) section. Supports figure 4. (A) Image-transcript overlays highlighting the spatial expression of key marker genes in each transcript colocalization

cluster. DAPI/WGA-AF488 composite in white, placed on a black background, different colours correspond to independent transcripts, each spot corresponds to one detected transcript. (B) Assignment of clusters to segmented cells based on Seurat single-cell analyses. DAPI/WGA-AF488 composite in white, placed on a black background, segmented cells in coloured lines, distinct colours for separate clusters. Arrows highlight transcript abundance heterogeneity between arbuscules at the same developmental stage. Scale bar, 100 µm.

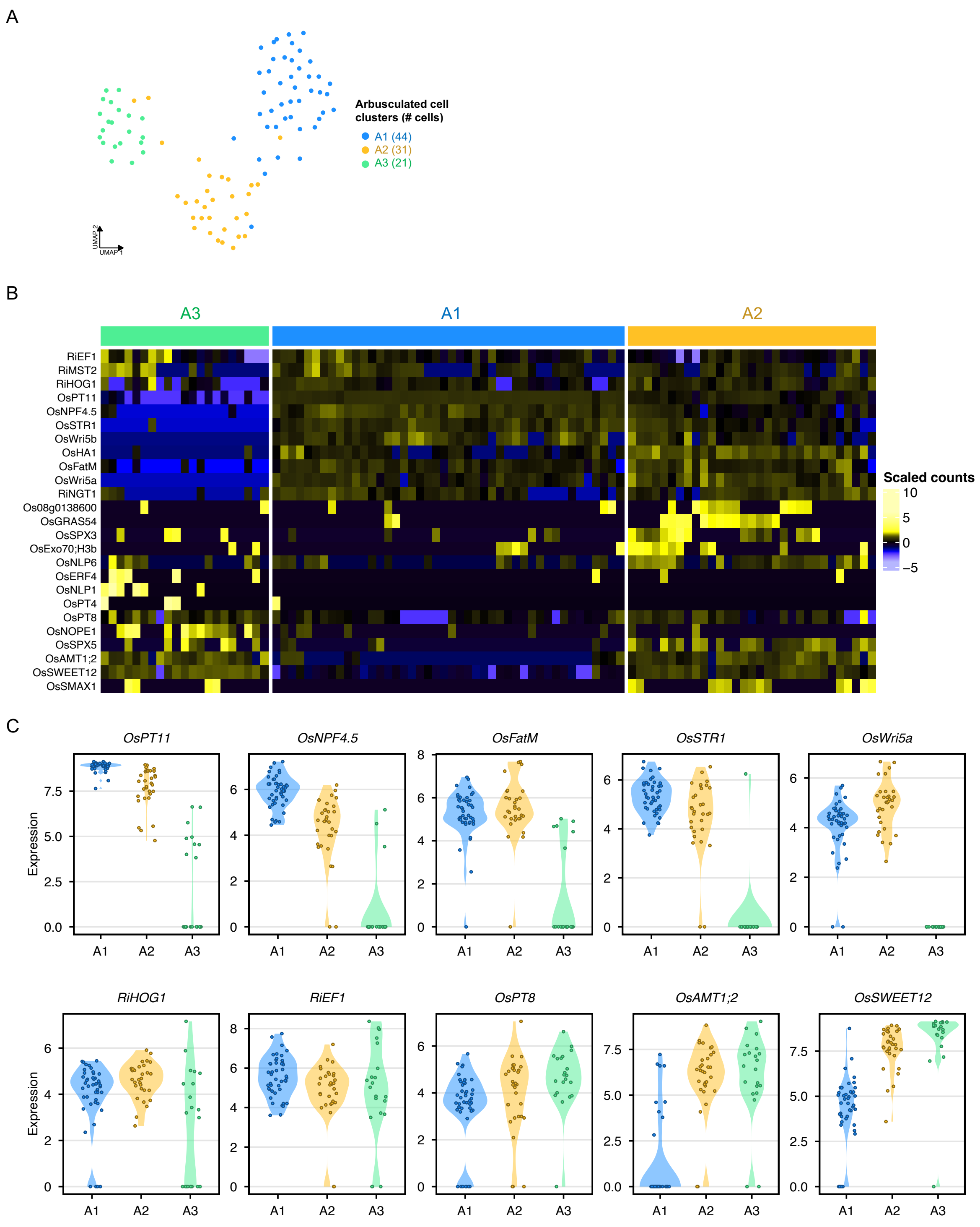

Supplementary Figure S9. Single-cell analysis for segmented arbusculated cells. Supports figure 4. (A) UMAP projection of segmented arbusculated cells, colour indicates the cluster (A1 to A3) assigned to each cell by *Seurat* single-cell analysis, number of cells in each cluster indicated in the legend. (B) Heatmap of normalised scaled transcript counts for each arbusculated cell (columns) for the top 10 marker genes (rows) in each arbusculated cell cluster (column sections), and their relative expression level compared to other clusters. (C) Expression level of a selection of top marker genes for arbusculated cell clusters, dots indicate individual arbusculated cells, expression levels are normalised based using *Seurat* default settings.

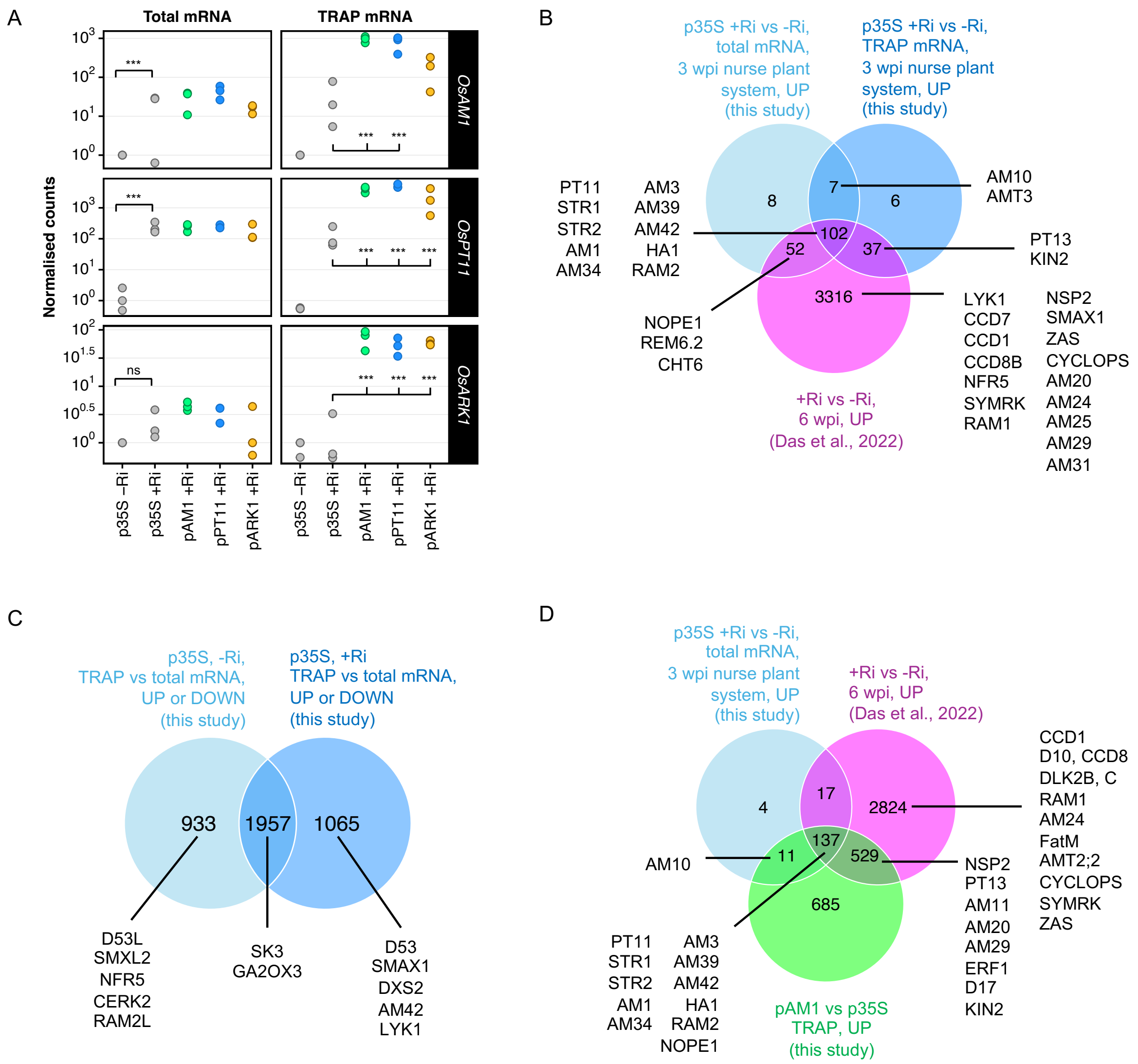

Supplementary Figure S10. Validation of AM-stage specific TRAP-seq of -Ri and +Ri roots. Supports figure 5. (A) Normalised gene counts for *OsAM1*, *OsPT11* and *OsARK1* in total and TRAP mRNA fractions for *p35S*, *pAM1*, *pPT11* and *pARK1:TRAP* lines. Significance levels as determined by DESeq2 shown with asterisks (* for p-value <0.05, ** for < 0.01, *** for < 0.001). (B) Venn diagram of overlaps between up-regulated genes for +Ri vs -Ri comparisons in this study for total and TRAP mRNA at three weeks post-inoculation (wpi) in a nurse plant set-up, with those from Das et al., 2022, at a later time-point (six wpi). Selected genes of interest highlighted. (C) Venn diagram of overlaps between enriched or depleted for TRAP vs total mRNA comparisons in non-inoculated (-Ri) or inoculated (+Ri) conditions. Selected genes of interest highlighted. (D) Venn diagram of overlaps between up-regulated genes for +Ri vs -Ri total mRNA comparisons in this study, those from Das et al., 2022, and enriched genes in *pAM1* vs *p35* TRAP mRNA. Selected genes of interest highlighted.

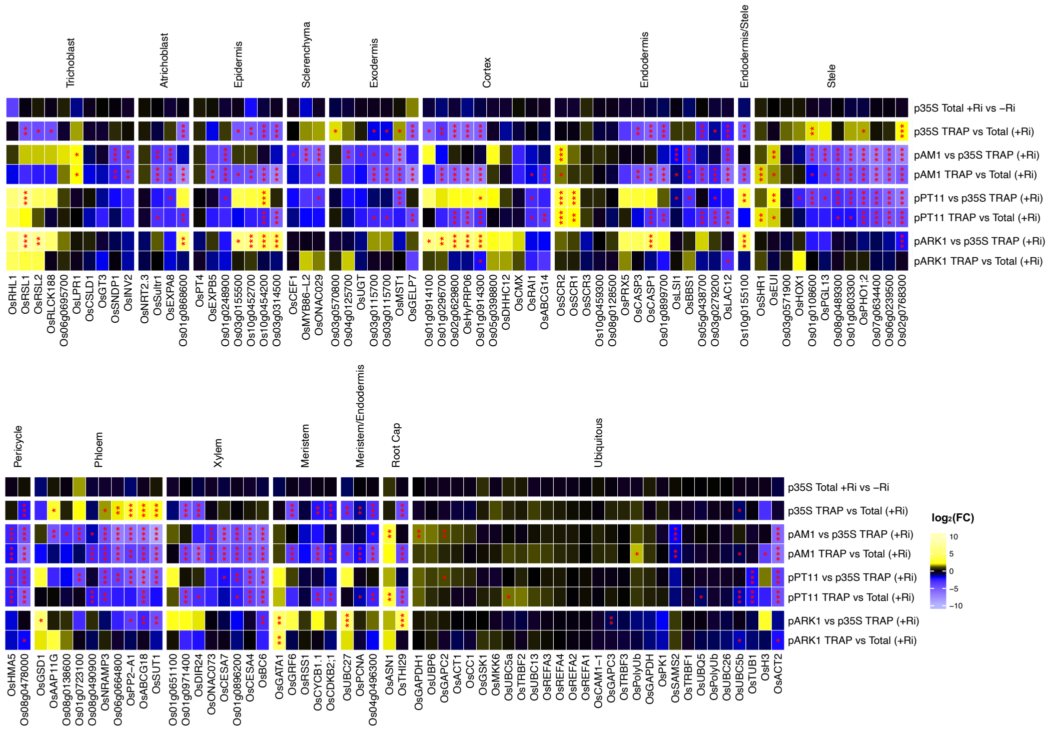

Supplementary Figure S11. Heatmap of $\log_2$FC for cell-identity markers. Supports figure 5. Predicted cell-type where expressed indicated above. Significance levels as determined by DGE using DESeq2 shown with asterisks (* for p-value <0.05, ** for < 0.01, *** for < 0.001). Genes were subjected to hierarchical clustering within each group. Gene IDs detailed in table S8.

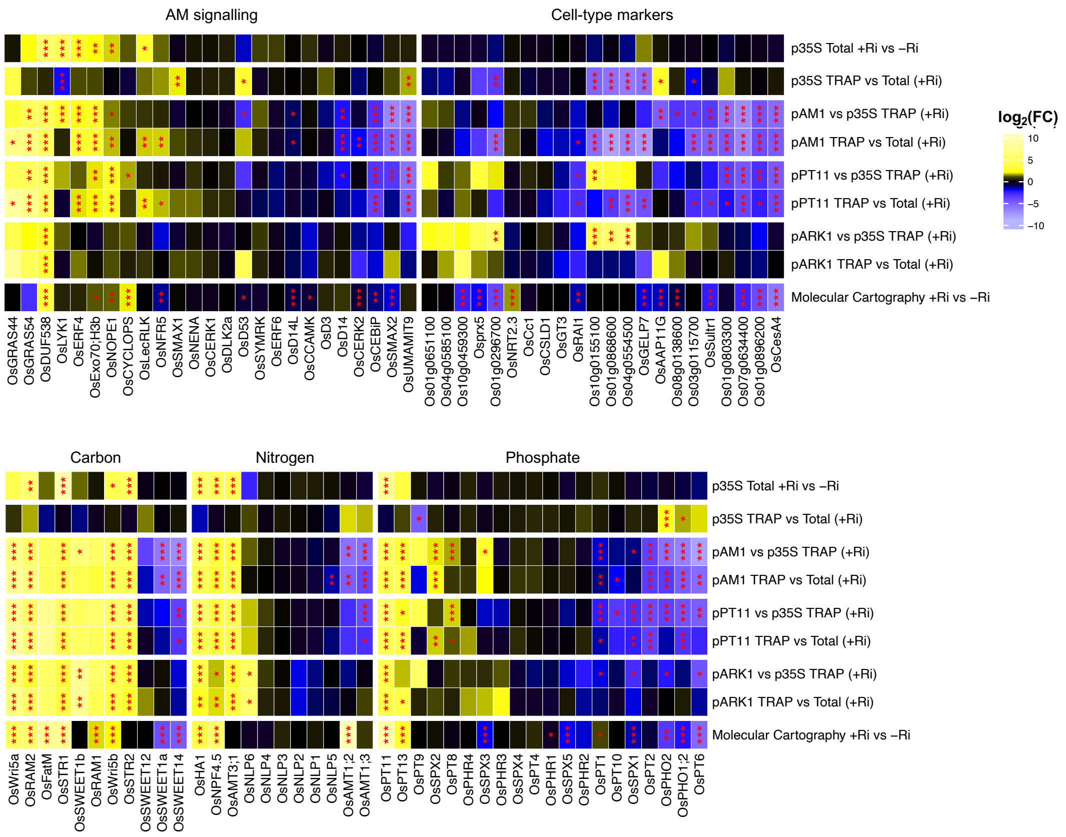

Supplementary Figure S12. Heatmap of log$_2$FC for genes in the Molecular Cartography probe panel. Supports figures 5 and 6. Also included are fold-changes of the +Ri vs -Ri comparison of spatial transcriptomics transcript spot count data. Significance levels as determined by DGE using DESeq2 shown with asterisks (* for p-value <0.05, ** for < 0.01, *** for < 0.001). Genes were subjected to hierarchical clustering within each group.

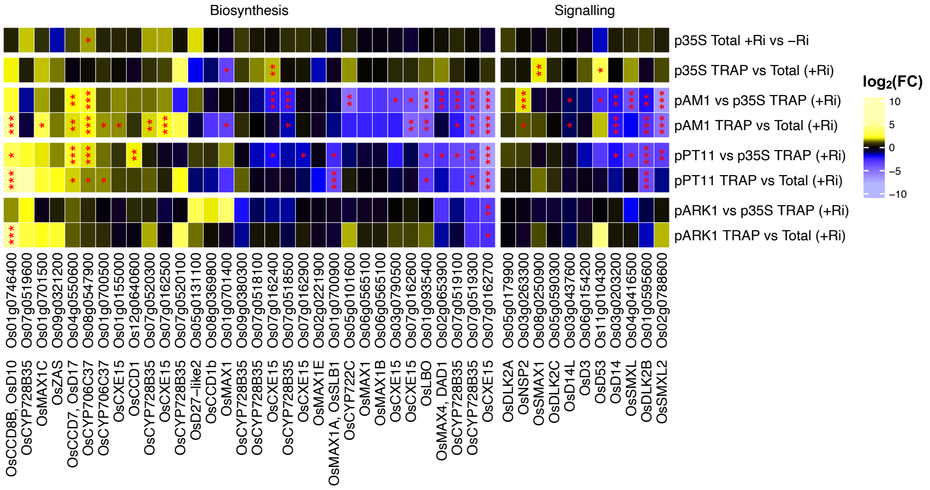

Supplementary Figure S13. Heatmap of $\log_2$FC for genes involved in strigolactone/apocarotenoid biosynthesis, perception and signalling. Supports figure 6. Significance levels as determined by DGE using DESeq2 shown with asterisks (* for p-value <0.05, ** for < 0.01, *** for < 0.001). Genes were subjected to hierarchical clustering within each group. Gene IDs listed in table S11.

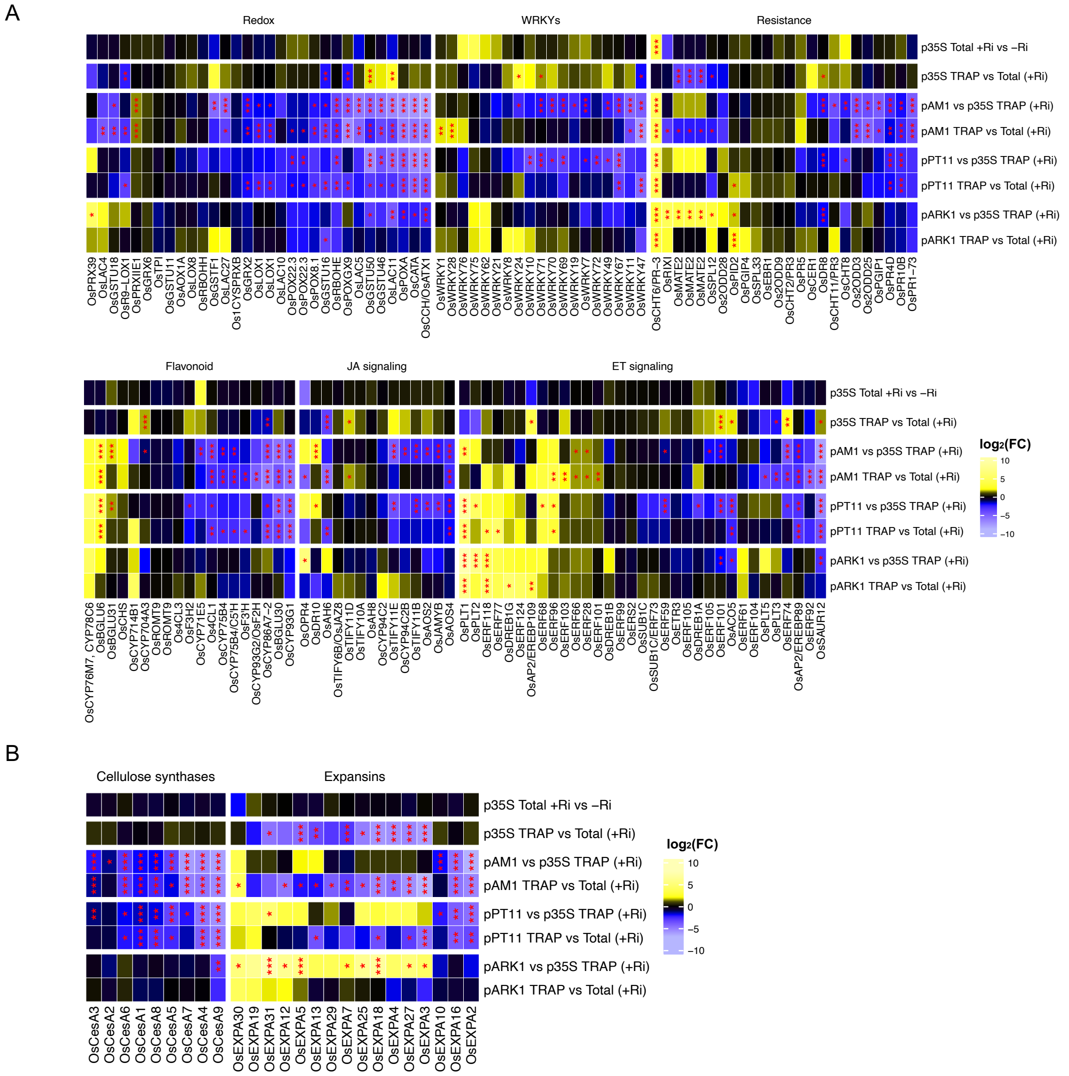

Supplementary Figure S14. Heatmap of log$_2$FC for a selection of genes related to various processes (indicated above) associated with immunity and defence, compiled by (Tang et al., 2021) and listed in table S12 (A), **cellulose synthases and expansins**, listed in table S13 (B). Supports figure 6. Significance levels as determined by DGE using DESeq2 shown with asterisks (* for p-value <0.05, ** for < 0.01, *** for < 0.001). Genes were subjected to hierarchical clustering within each group.

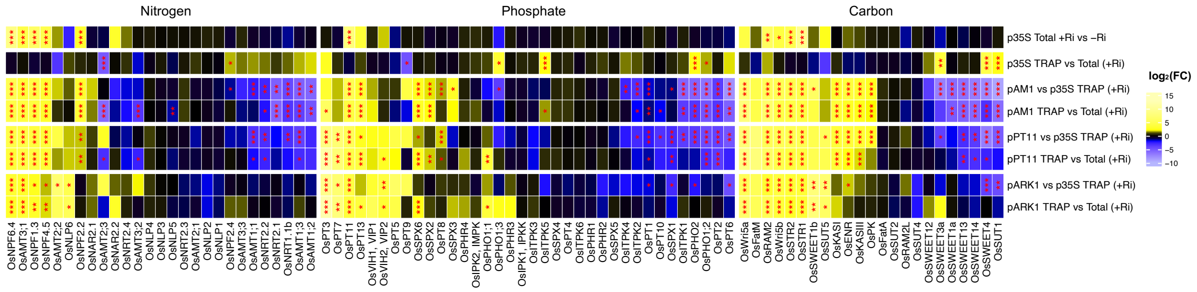

Supplementary Figure S15. Heatmap of log$_2$FC for key genes related to phosphate, nitrogen and carbon transport, perception and signalling. Supports figure 6. Significance levels as determined by DGE using DESeq2 shown with asterisks (* for p-value <0.05, ** for < 0.01, *** for < 0.001). Genes were subjected to hierarchical clustering within each group. Gene IDs listed in table S14.

References

Tang, B., Liu, C., Li, Z., Zhang, X., Zhou, S., Wang, G.-L., Chen, X.-L., & Liu, W. (2021). Multilayer regulatory landscape during pattern-triggered immunity in rice. *Plant Biotechnology Journal*, *19*(12), 2629–2645. https://doi.org/10.1111/pbi.13688

Das, D., Paries, M., Hobecker, K., Gigl, M., Dawid, C., Lam, H.-M., Zhang, J., Chen, M., & Gutjahr, C. (2022). PHOSPHATE STARVATION RESPONSE transcription factors enable arbuscular mycorrhiza symbiosis. *Nature Communications*, *13*(1), 477. https://doi.org/10.1038/s41467-022-27976-8
